# Assembly of a homohexameric minichromosome maintenance complex is dependent on ATP and DNA

**DOI:** 10.1016/j.jbc.2025.111072

**Published:** 2025-12-17

**Authors:** Oliver W. Noble, Clement Degut, Michael R. Hodgkinson, James P.J. Chong, Michael J. Plevin

**Affiliations:** 1 Department of Biology, University of York, York, UK; 2 York Structural Biology Laboratory, University of York, York, UK; 3 Centre of Excellence for Anaerobic Digestion, University of York, York, UK; 4 York Biomedical Research Institute, University of York, York, UK

**Keywords:** DNA helicase, minichromosome maintenance protein, DNA replication, complex assembly, structural biology

## Abstract

The minichromosome maintenance (MCM) complex is the replicative helicase in eukaryotes and archaea, unwinding genomic DNA upstream of DNA polymerase. The eukaryotic MCM complex forms from six different subunits (Mcm2–7), whereas in archaea, the complex is homohexameric. Both types of MCM can assemble into functional helicases *in vitro* in the absence of cofactors. However, despite being simpler in composition, we know little about how a homohexameric archaeal MCM assembles, largely because the field has lacked a convenient system to interrogate. Historically, characterization of archaeal MCMs has focused on proteins from thermophilic organisms, which typically form robust oligomers in solution. We have identified an uncharacterized MCM from the mesophilic archaeon *Mancarchaeum acidophilum* (*Mac*MCM) that shows strong DNA unwinding activity at room temperature. Unexpectedly, apo-*Mac*MCM is monomeric in solution, providing a first opportunity to investigate the mechanisms of assembly of an active homohexameric MCM complex *in vitro*. We show that *Mac*MCM requires both ATP and DNA to form an active homohexamer, and that the C-terminal winged-helix domain impedes oligomerization. We report the 3D structure of *Mac*MCM, which reveals similar numbers of interactions at subunit–subunit interfaces as eukaryotic MCMs but fewer than MCMs from thermophilic archaea. Finally, we show that installing subunit–subunit salt bridges from *Sulfolobus solfataricus* MCM into *Mac*MCM promotes oligomerization. Heterohexameric eukaryotic MCMs evolved from a homomeric ancestor. Our results identify structural and ligand-driven mechanisms of assembly that are conserved between homomeric and heteromeric MCMs.

DNA replication is an essential process for all living organisms. At the core of the replication machinery is a processive helicase that catalyzes strand separation of parental dsDNA ahead of DNA polymerase (1). Across the domains of life, two core replicative helicase families have evolved: archaea and eukaryotes utilize minichromosome maintenance (MCM) helicases (2), whereas bacteria use DnaB (3). Additional replicative helicases have also been characterized from viruses, notably g4 from bacteriophage T7 (4), gp41 from bacteriophage T4 (5), large T-antigen from simian vacuolating virus (SV-40 L-Tag) (6) and E1 helicase from papillomavirus (E1) (7).

All replicative helicases form ring-shaped hexamers and are believed to function by a steric exclusion model of unwinding (8). In this model, hydrolysis of nucleotide triphosphates (typically ATP) powers translocation of the helicase along a single strand of DNA that is enclosed within the central channel of the hexamer (8). The complementary strand is excluded from the channel, preventing reannealing of the parental strands. The direction of translocation along the fork (*i.e.*, the polarity of the helicase) is determined by the identity of the NTPase fold: a AAA+-type fold results in 3′-5′ translocation (MCM, SV-40 L-Tag, and BPV E1), whereas a RecA fold results in 5′-3′ translocation (DnaB, T7 g4, and T4 gp41) (8, 9).

Assembly of an active hexameric replicative helicase on dsDNA is a tightly controlled process *in vivo*, typically requiring numerous accessory factors (10). Replicative helicases unwind genomic dsDNA, and thus, loading must occur in the absence of free DNA ends, which precludes a mechanism in which the ring-shaped hexameric helicase is threaded onto DNA (9). *In vitro*, recombinantly produced hexameric replicative helicase can assemble spontaneously or in response to ligands. Viral helicases, such as E1, SV-40, T7 gp4, and T4 gp41, are predominantly monomeric in solution and form stable hexamers on addition of DNA, NTPs, or both (11, 12, 13, 14). Bacterial DnaB enzymes typically form stable hexamers in solution when magnesium is present (15).

Unlike all other replicative helicases, eukaryotic MCMs form a heteromeric complex (Mcm2–7), composed of six different subunits that assemble in a precise order (16). The parental ancestor of Mcm2–7 is believed to be a homohexameric MCM from archaea (17). Diversification of a single homomeric subunit into six different sequences introduces an opportunity for regulation in a more complicated cell cycle, for example, by providing subunit-specific phosphorylation sites (18). In the absence of accessory proteins, the core Mcm2–7 helicase can form an open ring structure, defined by a cleft or “gate” between subunits 2 and 5 (19). This structure can bind DNA in the presence of ATP and exhibits weak helicase activity (20, 21). The binding of the cofactors Cdc45 and GINS to form the Cdc45–Mcm2–7–GINS (or CMG) complex helps to close the 2 to 5 gate, resulting in a closed ring structure that shows markedly improved helicase activity (19, 22).

Much of the analysis of archaeal MCMs has focused on proteins that originate from thermophilic species. The common conclusion has been that MCMs from thermophilic archaea form robust higher order oligomers in solution under standard laboratory conditions. Indeed, a range of higher order oligomeric states has been reported for purified archaeal MCMs *in vitro*. Hexamers or dodecamers are most common in these systems (23, 24, 25, 26, 27), but stoichiometries, such as heptamers (28), octamers (29), and 14-mers, have also been reported (30), plus the oligomeric state observed appears to be heavily dependent on the temperature under which the experiments were performed (28, 30). Very few studies have reported evidence of monomeric archaeal MCM species in solution (30, 31). Where such species have been observed, the MCMs in question came from thermophilic organisms, and monomers were only observed at elevated temperatures. The current lack of a suitable system, whose transition from monomer to homohexamer can be practically characterized, has substantially limited our understanding of the molecular mechanisms underpinning assembly of homohexameric MCMs. We hypothesized that the historical focus on MCMs from thermophilic archaea has limited our ability to characterize MCM assembly because of the practical challenges associated with studying these processes at high temperature. Given that heterohexameric eukaryotic MCMs evolved from a homomeric ancestor, it is highly likely that core mechanisms of complex assembly are conserved and that many of these will be intrinsic properties of the basic MCM subunit. Discovery of a more experimentally tractable archaeal MCM would allow better biochemical characterization of the assembly of a homohexameric MCM and afford the chance to identify and evaluate conserved mechanisms.

We sought to address our limited appreciation of archaeal MCMs by screening a selection of previously unstudied examples from species that inhabit a broad selection of environmental niches. We postulated that organisms adapted to lower temperatures may have MCMs with assembly properties that were more amenable to characterization under ambient conditions. We identified an MCM from the mesophilic archaeon *Mancarchaeum acidiphilum* (*Mac*MCM) that has robust helicase activity at room temperature, but which predominates as a monomer in solution in the absence of ligands. This is the first time such a system has been reported, and its discovery has allowed us to conduct a detailed analysis of a homomeric MCM assembly process. Studying a mesophilic enzyme under ambient temperatures revealed assembly steps not previously seen for homohexameric MCMs and the importance of DNA and ATP in driving the association of monomeric MCM subunits. We elucidated the 3D structure of *Mac*MCM in a homohexameric state and determined that its subunit–subunit interfaces are more similar to heterohexameric eukaryotic MCMs than previously characterized homohexameric MCMs from thermophilic archaea. The discovery of *Mac*MCM permitted us to experimentally examine the assembly of a homohexameric MCM complex for the first time and to identify steps in this process that are fundamental and conserved across MCMs and other replicative helicases.

## Results

### An archaeal MCM with robust activity at ambient temperature

**Figure 1:**
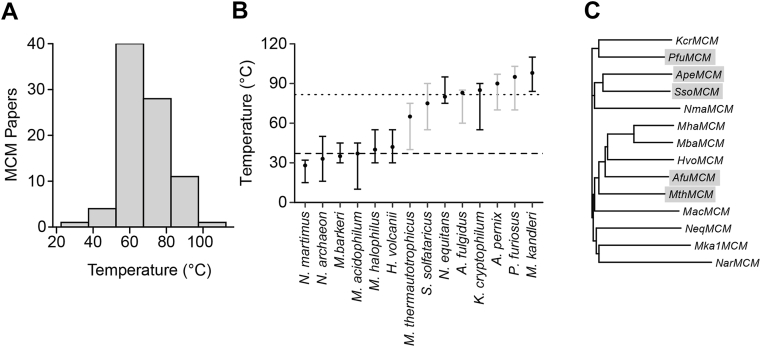
**Selection of a diverse library of 14 archaeal MCMs**. *A*, comparison of the number of publications released on archaeal MCMs against the natural temperature experienced by the enzyme *in vivo*. *B*, comparison of the preferred environmental temperatures of the organisms targeted. *Gray*, enzyme has been previously characterized; *black*, uncharacterized. Data points represent optimal growth temperature of organism; bars represent growth range. See Table S1 for supporting references. *C*, phylogram based on the sequence alignment of the 14 naturally occurring MCM sequences studied here. Sequences were retrieved from the KEGG database (27), and a phylogenetic tree was constructed using Clustal Omega (28). *Gray shading* indicates MCMs that have been previously characterized in the literature. KEGG, Kyoto Encyclopedia of Genes and Genomes; MCM, minichromosome maintenance.

Over 95% of studies of archaeal MCMs have focused on enzymes from organisms that occupy high-temperature environments (>65 °C; Fig. 1*A*). However, archaea inhabit a broad range of environments, which means that the proteins that perform their biochemistry have undergone environment-specific adaptations in their sequence while also retaining core function. To extend our understanding of the biochemistry of archaeal MCMs, we sought to characterize the activity of MCMs from a broader range of archaea (Fig. 1*B*; Table S1). We chose six MCMs from the genomes of mesophilic archaea (20–45 °C), which are adapted for life in various distinguishable habitats, including saline (*Nma*MCM), hypersaline (*Hvo*MCM*, Nac*MCM, and *Mha*MCM), anaerobic (*Mba*MCM), and acidic (*Mac*MCM). In addition, we sought to expand the number of MCMs from thermophilic archaea by selecting five examples (*Ape*MCM, *Afu*MCM, *Kcr*MCM, *Mka*MCM, and *Neq*MCM) from species that live in high-temperature environments but represent more distant phylogenetic lineages to well-studied systems (Fig. 1, *B* and *C*). Our analysis also included three well-studied MCMs from thermophilic organisms for which structures have been reported (*Mth*MCM, *Sso*MCM, and *Pfu*MCM). Certain archaea have parasitic or symbiotic relationships with other archaeal species. Of the 14 selected, three come from parasitic/symbiont archaea (*Nac*MCM, *Neq*MCM, and *Mac*MCM), all of which have extremely small genomes (<1 Mb) that lack genes encoding core life processes and as such survive *via* an obligate interaction with another archaeal species. Bioinformatic analyses were performed on each sequence to confirm the presence of conserved subdomains and motifs within each MCM (Figs. S1 and S2) (32, 33, 34). To complete our selection, we included a previously engineered chimeric MCM, generated by fusing the N-terminal domain (NTD) of *Sso*MCM with the ATPase domain of *Pfu*MCM (*Sso*_*N*_*Pfu*_*C*_MCM) (35), as well as the reverse chimera, *Pfu*_*N*_*Sso*_*C*_MCM. The C-terminal winged-helix domain (WHD) was not included in either chimeric enzyme.

Recombinant His_10_-MCMs were overexpressed in *Escherichia coli*, and protein expression and solubility were assessed using gel electrophoresis (Fig. S3). While expression and solubility levels varied considerably, bands at molecular weights (MWs) consistent with at least 11 of the MCM targets were observed in the soluble fraction following centrifugation of cell lysates. In general, MCMs from thermophilic organisms expressed at higher levels and were more soluble than MCMs from mesophilic organisms. For preliminary characterization, each MCM was purified using a single immobilized metal affinity chromatography step. Different ranges of sample purity and nucleic acid contamination were observed for each MCM construct, but predominant bands consistent with the expected MW were observed in elution fractions for 13 of the 16 samples; only *Ape*, *Mha*, and *Nac* showed insufficient levels of expected protein (Fig. S4; Table S2).

**Figure 2:**
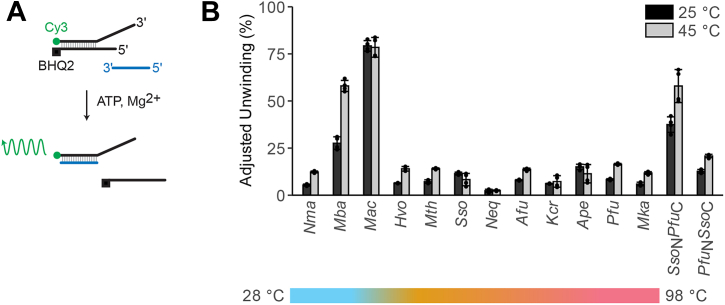
**Characterization of DNA unwinding of 14 archaeal MCM helicases**. *A*, overview of the FRET-based biochemical activity screen (30). Protein is pre-equilibrated with a forked DNA substrate. Addition of ATP–Mg^2+^ initiates substrate unwinding, which spatially separates a fluorophore (Cy3) and quencher (BHQ2) causing an increase in fluorescence (λ = 570 nm). A scavenger strand (*blue*) prevents reannealing. *B*, percentage DNA unwound by each MCM sample at 25 °C (*black*) and 45 °C (*gray*) after 30 min. Unwinding was quantified by subtracting a no helicase control and then standardizing against a maximum fluorescence well, containing nonannealed Cy3-labeled ssDNA. Bars represent mean unwinding (n = 4). Error bars correspond to ±1 SEM. MCM, minichromosome maintenance.

A fluorescence-based dsDNA unwinding assay (36) was used to assess the helicase activity of each protein (Fig. 2*A*). For comparison, unwinding values were standardized to protein concentration (unwinding % per 1000 nM hexamer). All samples tested exhibited at least a small degree of substrate unwinding under the assay conditions (Fig. 2*B*; Fig. S5). Of the nonsynthetic enzymes, the two most active at 25 °C were from mesophilic organisms (*Mac*MCM and *Mba*MCM). *Sso*_*N*_*Pfu*_*C*_MCM showed a similar degree of unwinding to *Mba*MCM, but the synthetic construct lacks a regulatory WHD, which has been shown to have a negative effect on unwinding rate in MCMs (37, 38).

MCMs from thermophilic archaea, including *Mth*, *Pfu*, and *Sso*MCM, showed only low activity at 25 °C. Increasing the assay temperature to 45 °C improved activity for most samples tested, but, even at the elevated temperature, none was more active than *Mac*MCM. *Mba*MCM also demonstrated high activity at both 25 and 45 °C; however, as expression yields of *Mba*MCM were considerably lower than *Mac*MCM (6 *versus* 92 mg protein per liter of culture), we elected to take forward the latter for further study. All further biochemical characterizations were performed with samples that had been subjected to additional purification steps (Fig. S6).

### *Mac*MCM homohexamer unwinds DNA with sigmoidal kinetics

**Figure 3:**
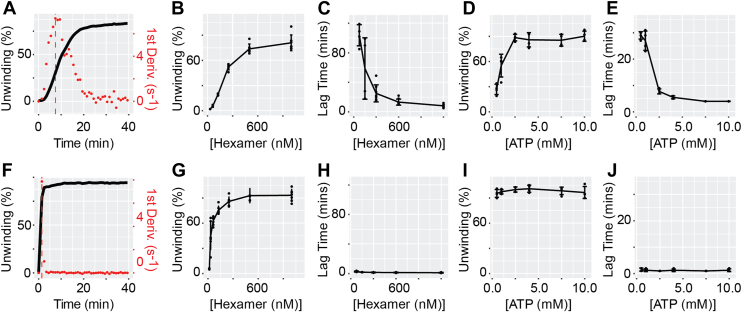
**Interactions with protein, ATP, and the winged-helix domain (WHD) influence a slow kinetic step for *Mac*MCM assembly**. Assay data and analysis for (*A*–*E*) *Mac*MCM and (*F*–*J*) *Mac*MCM^ΔWHD^. *A* and *F*, example of lag time calculation for a real-time helicase trace for *MacMCM*. The first derivative is calculated from an experimental unwinding curve, and the time taken to reach the maximum rate is extracted (*red dotted line*). *B* and *G*, net unwinding data for *Mac*MCM measured against protein concentration. *C* and *H*, lag time extracted from each protein concentration in part (*B*). *D* and *I*, net unwinding data for *Mac*MCM measured against ATP concentration. *E* and *J*, a “lag time” extracted from each ATP concentration in part *D* and *I*. Concentration when reagent was fixed: MCM hexamer, 1 μM; forked DNA substrate, 50 nM; ATP and MgCl_2_, 4 mM and 10 mM, respectively. Bars represent mean unwinding (n = 4). Error bars correspond to ±1 SEM. *Mac*MCM, MCM from *Mancarchaeum acidophilum*; MCM, minichromosome maintenance.

Analysis of the dsDNA unwinding properties of *Mac*MCM revealed an unexpected sigmoidal profile, with a slow initial rate that increases to a maximum after ∼6 min when measured at 25 °C (Fig. 3*A*). By contrast, *Sso*_N_*Pfu*_C_MCM exhibited more standard reaction kinetics (Fig. S7). Sigmoidal enzyme kinetics suggest the presence of a primary, rate-limiting step that precedes DNA unwinding. Such a step has been observed for Mcm2–7, where slow nucleotide-dependent changes occur on a time scale of 5 to 10 min to permit stable assembly of the full hexameric complex on DNA. An equivalent behavior has not previously been reported for a homohexameric MCM.

To determine which factors influence the activity of *Mac*MCM, DNA unwinding assays were performed using a series of different protein and ATP concentrations. To quantify the sigmoidal kinetics of *Mac*MCM, the first derivative of the measured unwinding curve was calculated, and from that, the time taken to reach the maximum unwinding rate was determined (Fig. 3*A*). We refer to this metric as “lag time.”

Decreasing the concentration of *Mac*MCM results in lower relative enzymatic activity and a longer lag time (Fig. 3, *B* and *C*). The effect of ATP on lag time was also measured against a physiological range of ATP concentrations (0.5–10 mM). Reducing the ATP concentration from 2.5 to 0.5 mM markedly decreased net enzyme activity and extended the lag time toward the lower time limit of the assay (Fig. 3, *D* and *E*). Together, these results show that both protein–protein and protein–ATP interactions influence the lag-phase kinetics of *Mac*MCM. The observation that higher concentrations of either ATP or protein decrease the lag time suggests that this phenomenon is related to an association event, for example, the assembly of a macromolecular MCM complex on DNA.

### Sigmoidal kinetics of *Mac*MCM are influenced by the WHD

Cryo-EM structures of both Mcm2–7 and CMG complex show the Mcm5 WHD occupying a position in the central channel of the heterohexamer (39, 40). One possibility is that the lag time in activity observed for *Mac*MCM *in vitro* relates to the movement of the WHD in response to both nucleotide and DNA. To assess this, we evaluated the DNA unwinding properties of a truncated variant of *Mac*MCM that lacks the WHD (*Mac*MCM^ΔWHD^) across a range of protein and ATP concentrations (Fig. 3, *F*–*J*). Consistent with studies of other MCMs (37, 38), removal of the WHD of *Mac*MCM resulted in substantially elevated activity, with close to 100% of the substrate unwound within the first few minutes (Fig. 3*F*). Moreover, the *Mac*MCM^ΔWHD^ construct did not exhibit a measurable lag time at any of the protein or ATP concentrations tested (Fig. 3, *H* and *J*). These data show that removal of the WHD either eliminates the lag time or substantially reduces it beyond the detection limit of our assay. Nevertheless, a plausible explanation is that the WHDs of *Mac*MCM are directly involved in the rate-limiting step in a similar manner to that which may occur for Mcm2–7 *in vitro* (20, 41).

### Both ATP and DNA are required for *Mac*MCM to form a homohexamer

Previously studied archaeal MCMs form stable oligomers in solution (typically hexamers or dodecamers) even in the absence of ATP or DNA (23, 24, 25, 26, 27, 28, 29, 30). To evaluate the oligomeric state of *Mac*MCM in the presence of DNA, we incubated the helicase with different ligands before subjecting the samples to analytical size-exclusion chromatography (SEC). ssDNA was used in these experiments to allow us to characterize complexes formed on DNA in the absence of any unwinding.

**Figure 4:**
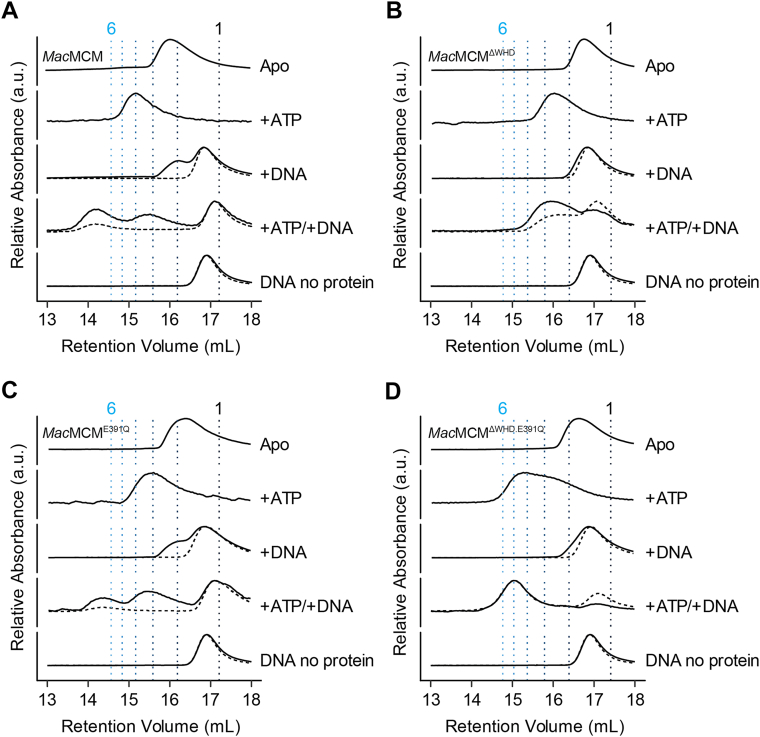
**ATP and DNA contribute to stable hexamer formation by *Mac*MCM**. The loading of *Mac*MCM constructs onto DNA was analyzed by analytical size-exclusion chromatography (SEC). Protein samples (10 μM) were preincubated with or without an equimolar ratio of fluorescein-labeled ssDNA substrate (polyT_50_) before application to a Superose 6 Increase 10/300 GL SEC column. Where stated, ATP at 1 mM and Mg^2+^ at 10 mM were added to the buffer. UV absorbance was monitored at both 290 nm (*solid trace*) and 495 nm (*dotted trace*). *Vertical dotted lines* indicate the expected elution volumes of MCM oligomers with one to six subunits. *A*, *Mac*MCM; *B*, *Mac*MCM^ΔWHD^; *C*, *Mac*MCM^E391Q^; and *D*, *Mac*MCM^E391Q.ΔWHD^. *Mac*MCM, MCM from *Mancarchaeum acidophilum*; MCM, minichromosome maintenance; WHD, winged-helix domain.

Without ATP or ssDNA, *Mac*MCM and *Mac*MCM^ΔWHD^ eluted at larger volumes than would be expected for a homohexamer (Fig. 4, *A* and *B*). This was further confirmed using SEC–multiangle laser light scattering (MALLS), which showed that both constructs eluted with MWs closer to a monomer, and that the MW calculated was dependent on the concentration of protein loaded (Fig. S8). This latter observation suggests that apo *Mac*MCM exists in a monomer–oligomer equilibrium in solution and that the monomeric species predominates at lower protein concentrations and in the absence of ligands. These results also suggest that the core region of *Mac*MCM, comprising NTD and AAA+ domains, is itself unable to form a stable homohexamer in solution. This contrasts the *Mac* enzyme with previously characterized MCMs from (hyper)thermophilic archaea, including *Ape* (23), *Sso* (38), and *Afu* (23), all of which have been shown to form homohexamers in solution in the absence of ATP and DNA.

The elution profile of *Mac*MCM depends on which ligands are present (Fig. 4). The addition of ssDNA does not change the elution volume of either *Mac*MCM or *Mac*MCM^ΔWHD^ or of the ssDNA ligand. In the presence of ATP–Mg^2+^, both *Mac*MCM and *Mac*MCM^ΔWHD^ show a slight decrease in elution volume, but the size of the change does not support the formation of a homohexamer. Only when both ssDNA and ATP are present does the elution profile of *Mac*MCM show a species that elutes at a volume more consistent with a homohexamer (Fig. 4*A*). Moreover, only in the presence of ATP does *Mac*MCM coelute with ssDNA. By contrast, when *Mac*MCM^ΔWHD^ is mixed with ssDNA and ATP, the elution volume decreases but not by the same degree as *Mac*MCM. As the ΔWHD variant is considerably more active than the wildtype *Mac*MCM (Fig. 3*F*), we hypothesize that higher enzymatic turnover of ATP during the SEC experiment reduces the lifetime of a full *Mac*MCM^ΔWHD^–ATP–ssDNA complex such that a hexameric MCM–DNA species is not resolved. To evaluate the impact of ATP hydrolysis, SEC experiments were repeated using equivalent Walker B mutation (E391Q) constructs that render the ATPase domain inactive but that should still permit ATP binding (42) (Figs. S6 and S9). In the presence of ATP, but with hydrolysis no longer possible, both *Mac*MCM^E391Q^ and *Mac*MCM^ΔWHD.E391Q^ coeluted with ssDNA at elution volumes consistent with a homohexamer (Fig. 4, *C* and *D*).

### ATP turnover promotes assembly of a DNA-bound full-length *Mac*MCM homohexamer

Hexamerization is a requirement for stable binding of DNA by MCMs (43). In agreement with previous studies, we found that *Sso*_*N*_*Pfu*_*C*_MCM is an obligate homohexamer at room temperature and pressure (Fig. S8) and that it binds forked DNA in the absence of nucleotide cofactors (*K*_*d*_ = 60 ± 10 nM; Table 1; Figs. S10, S11). Adding ATP increases DNA-binding affinity threefold to 20 ± 1 nM compared with apo *Sso*_*N*_*Pfu*_*C*_MCM. Incubating *Sso*_*N*_*Pfu*_*C*_MCM with either nonhydrolyzable ATP analogs (AMP–PCP, which mimics the prehydrolysis state; or ADP–AlF_4_^-^, which mimics the transition state) or ADP did not significantly affect the affinity for DNA.

**Table 1:** DNA binding affinity of MCM constructs is differentially impacted by nucleotides

Ligand	E418Q	State	*K*_*d*_ (nM)		
			*Mac*MCM	*Mac*MCM^ΔWHD^	*Sso*_N_*Pfu*_C_MCM
—	×	No nucleotide	480 ± 35	>1000 nM	60 ± 10
AMP–PCP	×	Prehydrolysis	700 ± 70	>1000 nM	55 ± 4
ATP	✓	Prehydrolysis	780 ± 8	114.3 ± 5.4	ND
ADP–AlF_4_	×	Transition state	660 ± 60	>1000 nM	45 ± 6
ADP	×	Posthydrolysis	570 ± 60	>1000 nM	55 ± 2
ATP	×	Active hydrolysis	120 ± 8	60 ± 2	20 ± 1

Binding affinities were measured *via* fluorescence polarization using a fluorescently labeled forked DNA substrate in the absence of the presence of nucleotides that mimic steps of the catalytic cycle.

Reactions were conducted using 1 nM DNA and 4 mM nucleotide plus 10 mM MgCl_2_ when added. Error represents ±1 standard error of the *K*_*d*_ with *N* = 3.

ND, not determined.

Compared with *Sso*_*N*_*Pfu*_*C*_MCM, *Mac*MCM binds to a forked DNA substrate with more moderate affinity in the absence of ATP (*K*_*d*_ = 480 ± 35 nM). Addition of AMP–PCP, ADP–AlF_4_^-^, or ADP slightly reduced DNA binding affinity compared with apo-*Mac*MCM (Fig. S10). The Walker B mutant of *Mac*MCM (E391Q), which does not turn over ATP, interacts with DNA with an affinity of 780 nM in the presence of ATP. However, a large increase in affinity for DNA is seen when the enzyme is capable of turning over ATP (*K*_*d*_ = 115 ± 8 nM). This represents a fourfold change compared with *Mac*MCM in the absence of ATP and an eightfold change compared with the E391Q variant in the presence of ATP.

Truncation of the WHD of MacMCM decreased affinity for DNA in all conditions tested, except for when ATP was present (Table 1). Apo-*Mac*MCM^ΔWHD^ bound DNA with much weaker affinity compared with the apo full-length protein (Table 1; Fig. S10). Likewise, weak binding to DNA was seen in the presence of AMP–PCP, ADP–AlF_4_^-^, or ADP. However, compared with wildtype *Mac*MCM, a smaller twofold difference in affinity for DNA was seen between active (*K*_*d*_ = 60 ± 2 nM) and catalytically inactive (*K*_*d*_ = 115 ± 5 nM) variants of *Mac*MCM^ΔWHD^ when ATP is present. In both cases, the affinity of the WHD truncation for DNA is higher than the full-length *Mac*MCM.

### The homohexameric structure of *Mac*MCM resolved at 2.6 Å

The unexpected sigmoidal kinetics and self-association properties observed for *Mac*MCM suggested that there may be differences in the 3D structure of *Mac*MCM compared with previously determined 3D structures of MCMs from thermophilic archaea. We conducted crystallization screens using various *Mac*MCM constructs. Crystals that diffracted to 2.6 Å were obtained using a construct that lacked the C-terminal WHD and carried a point mutation in the Walker B motif (E391Q) to render it inactive. Crystals grew in the presence of ATP and MgCl_2_ over a period of 3 days.

**Figure 5:**
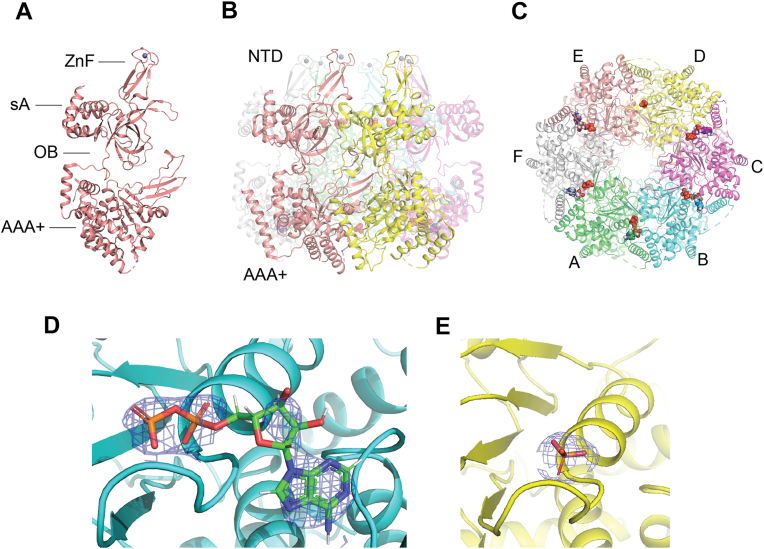
**The crystal structure with 2.6 Å resolution of the core *Mac*MCM^ΔWHD E391Q^ hexamer bound to ADP and phosphate**. *A*, view of a single MCM subunit (chain E), showing the positions of subdomain A (sA), zinc finger (ZnF), oligosaccharide/nucleotide binding fold (OB), and ATPase associated with various cellular activities (AAA+) domain. *B*, view perpendicular to the central channel, where the N-terminal domain (NTD) and C-terminal domain (CTD) tiers are clearly defined. *C*, view into the central channel from the CTD side. The position of ADP and phosphate is represented in the *sphere format*. *D*, close-up view of one of the five ADP molecules modeled in an AAA+ active site. ADP is shown in the *stick format*, and electron density (Omit-map) is shown in the *mesh format*. *E*, close-up view of the phosphate ion modeled in the active site at the interface of subunits *D* and *E*. Phosphate is shown in *stick format*, and electron density (Omit-map) is shown in *mesh format*. All images were prepared using PyMol (19). Each MCM subunit is colored as stated and represented in the *cartoon format*. *Mac*MCM, MCM from *Mancarchaeum acidophilum*; MCM, minichromosome maintenance; WHD, winged-helix domain.

The asymmetric unit contained a single ring-shaped *Mac*MCM^ΔWHD,E391Q^ homohexamer (Table S3). Each monomer is composed of two modular domains: an N-terminal DNA-binding domain (NTD) and a C-terminal ATPase domain (AAA+; Fig. 5). The native linker joins the NTD and AAA+ domains, which in some of the previous archaeal MCM crystal structures was modified to generate a successful crystallization construct (44). The NTD is further divisible into three subdomains, which consist of a four-helix bundle (sA), a four cysteine (C_4_)–type zinc finger (ZnF), and an oligonucleotide binding fold (OB-fold) (45). The active sites of the ATPase domain are formed at subunit–subunit interfaces with both subunits contributing residues, as is typical in MCMs (46). In each ATPase site, the Walker A, B, and sensor-1 motifs are provided by the *cis-*acting subunit, whereas the arginine finger and sensor-2 motifs are provided by the *trans*-acting subunit. The conserved DNA-binding hairpins are found in the central channel with each subunit providing three hairpins: the N-terminal β-hairpin, the helix-2 insert, and the pre–sensor-1 β-hairpin (45, 47, 48).

Four classes of ligand were observed in the structure: Zn^2+^ ions were identified in all six ZnFs; ADP was identified in five of the six ATPase active sites; a phosphate ion was identified in the remaining ATPase active site (formed by subunits D and E); and six phosphate ions were coordinated by each OB-fold (Fig. 5). No density was observed in the ATPase active site for Mg^2+^ or for the γ-phosphate of ATP. Although the *Mac*MCM^ΔWHD-E391Q^ construct used for crystallization showed no ATPase activity in solution, it does not rule out the possibility of spontaneous ATP hydrolysis during crystallization or residual levels of ATP hydrolysis activity that would be too low to measure in our NMR assay. Despite the presence of two distinct classes of ligands in the ATPase sites, the structures of the active sites are largely identical (Fig. S12), resulting in the DNA-binding hairpins adopting a planar orientation with respect to the tiers of the hexamer. The largest topological difference between *Mac*MCM and other MCM structures concerned the position of the ZnFs. The distance between neighboring ZnFs in *Mac*MCM is on average further apart than has been observed in other homomeric MCM hexamer structures (Fig. S13).

Overall, the 3D structure of *Mac*MCM is consistent with previously published hexameric archaeal and eukaryotic MCMs (Figs. S14, S15). When each subunit of *Mac*MCM is superposed in turn with each subunit in a homohexameric MCM (*Sso*MCM, Protein Data Bank [PDB] code: 6MII) (44) or a Mcm2–7 heterohexamer (*Sce*Mcm2–7, PDB code: 6EYC) (49, 50), the average all-atom RMSD ± 1 standard deviation is 2.5 ± 0.2 Å and 3.3 ± 0.8 Å, respectively. At the level of domains, the largest differences are found at the NTD, where the structure of *Mac*MCM is more similar to *Sso*MCM (average RMSD: 2.5 ± 0.2 Å) than to *Sce*Mcm2–7 subunits (average RMSD: 3.5 ± 1.6 Å). By comparison, the highly conserved C-terminal ATPase domain shares excellent and more consistent structural homology with both *Sso*MCM (average RMSD: 1.3 ± 0.1 Å) and *Sce*Mcm2–7 (average RMSD: 1.8 ± 0.2 Å).

### *Mac*MCM is adapted to ambient temperature environments

**Figure 6:**
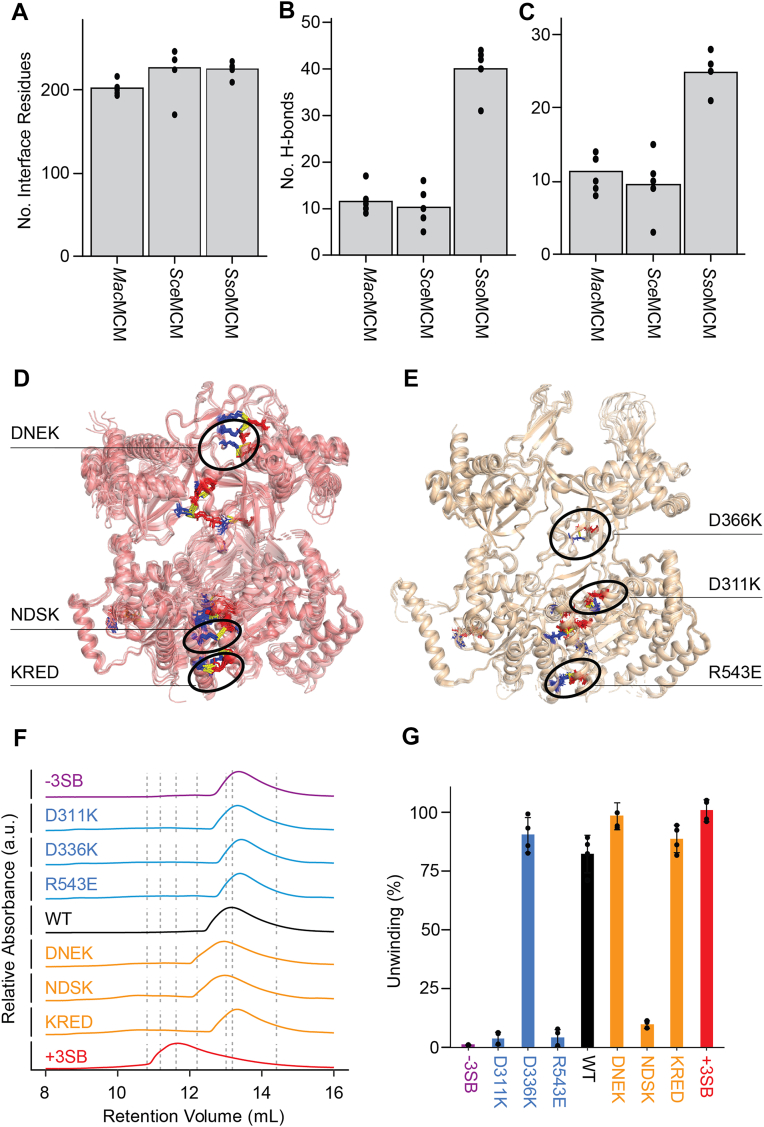
**Salt bridges are essential for functional *Mac*MCM oligomer formation**. *A*–*C*, 3D structures of different MCM hexamers (*Mac*MCM—PDB code: 8Q67, *Sce*MCM—PDB code: 6EYC, and *Sso*MCM—PDB code: 6MII) were examined using PDBePISA. Average number of (*A*) residues, (*B*) hydrogen bonds, and (*C*) salt bridges at subunit–subunit interfaces. *Black points* represent raw values for each subunit–subunit interface. *D* and *E*, location of selected salt bridges at subunit–subunit interfaces of (*D*) *Sso*MCM and (*E*) *Mac*MCM. *F*, *Mac*MCM mutants were examined by analytical size-exclusion chromatography (SEC). Protein samples (10 μM) were applied to a Superdex S200 Increase 10/300 GL SEC column. UV absorbance was monitored at 280 nm. *Vertical dotted lines* indicate the expected elution volumes of MCM oligomers with one to six subunits. *G*, percentage of dsDNA unwound by each MCM mutant was determined at 25 °C after 30 min. Bars represent mean unwinding (n = 4). Error bars correspond to ±1 SD. *Mac*MCM, MCM from *Mancarchaeum acidophilum*; MCM, minichromosome maintenance; PDB, Protein Data Bank.

Our interest in *Mancarchaeum acidophilum* came from a desire to characterize an MCM from a mesophilic archaeon. *M. acidophilum* was first discovered in Anglesey in Northwest Wales, and consequently, this organism will infrequently experience temperatures exceeding 25 °C. Enzymes from thermophilic organisms typically contain a higher number of salt bridge interactions, which have been proposed to increase stability at higher temperatures (51, 52). By contrast, at lower temperatures, larger numbers of salt bridges may impede necessary conformational change and thus activity. The spiral staircase model of DNA translocation by MCMs requires substantial conformational change between subunits in the hexameric ring (44). Our analysis of *Mac*MCM reveals significant differences in its oligomerization properties to other thermophilic MCMs, and so to better understand the relatively weak association of *Mac*MCM subunits at ambient conditions, we compared subunit interfaces from the hexameric MCM structures of *Sso*MCM (PDB code: 6MII) (44), *Sce*Mcm2–7 (PDB code: 6EYC) (49, 50) and *Mac*MCM (this study) using Proteins Interfaces Structures and Assemblies (53). Despite the primary structure of each eukaryotic *Sce*Mcm2–7 subunit having on average 31% more amino acids than *Sso*MCM or *Mac*MCM, the oligomerization interfaces of all three MCMs are formed from equivalent numbers of residues. However, subunit–subunit interfaces in both *Sce*Mcm2–7 and *Mac*MCM possess 25% fewer hydrogen bonds and half the number of salt bridges compared with *Sso*MCM (Fig. 6abc). While limited by the availability of suitable 3D structures for archaeal hexameric MCMs, this analysis is consistent with adaptation of *Sso*MCM to high-temperature environments involving an increased number of subunit–subunit interactions (51).

To further evaluate the role of noncovalent interactions in the formation of MCM oligomers, we identified salt bridges that were either conserved or lost between *Mac*MCM and *Sso*MCM (Figure 6de, Fig. S16). Although salt bridges are less abundant, on a per-interaction basis, they contribute more to protein stability compared with hydrogen bonds, therefore offering a more efficient target for probing interfaces. We generated a series of *Mac*MCM mutants that focused on intersubunit salt bridges. Mutations were carefully chosen to be greater than 10 Å from the nucleotide active site to minimize impact on ATP binding and catalysis. We generated three charge swap “minus-SB” mutants (D311K, D366K, and R543E) at specific intersubunit salt bridge pairs within our *Mac*MCM structure as well as a triple site mutation that comprised all three individual minus-SB variants. We then compared the *Mac*MCM structure with *Sso*MCM to identify three “plus-SB” sites, where we hypothesized that salt bridges could be restored (DNEK, D59N–E61K; KRED, K476R–E537D; NDSK, and N484D–S532K) (Fig. S16). The DNEK plus-SB mutation installs a lysine at position 61, which should form a salt bridge with E167. We added a D59N mutation as our structure suggests that D59 may impede the formation of a salt bridge between E167 and the mutant lysine at position 61. A construct comprising all three plus-SB mutants was also generated. All plus- and minus-SB variants were produced to equivalent purity (Fig. S17) before being subjected to further biochemical interrogation.

All four minus-SB mutants exhibited a slight increase in elution volume on SEC, suggesting a shift in the oligomerization equilibrium toward a monomeric species (Fig. 6*F*). With the exception of KRED, the single-site plus-SB mutants exhibited a slight decrease in elution volume, whereas the triple-site plus-SB mutants resulted in a large 1.5 ml shift, consistent with a shift toward a larger oligomeric species. Helicase assays were used to evaluate whether the plus- and minus-SB mutants impacted DNA unwinding activity. All minus-SB mutants, except D366K, reduced activity (Fig. 6*G*), suggesting that the salt bridges observed in our crystal structure contribute to the formation and/or stabilization of a functional homohexamer. Other than NDSK, which showed no activity, plus-SB mutants resulted in a slight increase in activity. Interestingly, the net result of combining all plus-SB mutations was dominant over the reduction in activity observed for the site NDSK alone (Fig. 6*F*). In all instances, the mutations did impact the lag time seen in unwinding assays (Fig. S18). This observation is consistent with SEC results, which showed that none of the plus-SB mutations converted *Mac*MCM into an obligate hexamer.

## Discussion

All replicative helicases are believed to unwind DNA as ring-shaped hexamers. The MCM family is unique in containing helicases that function as both homohexamers and heterohexamers. In the assembly pathway of the heterohexameric MCM complex, each subunit has evolved to play a different role. By contrast, self-association of six identical subunits into a homohexameric MCM imposes symmetry-induced restraints as all subunits have the same sequence and structure. Despite considerable recent advances in our understanding of the assembly of the eukaryotic Mcm2–7 complex, we still have few details concerning how archaeal MCMs self-associate, the role of ATP and DNA in the assembly pathway, and ultimately what features are common to both eukaryotic and archaeal MCM subunits and what differences have evolved. Our appreciation of the biochemistry and structural biology of archaeal MCMs is largely limited to examples from two thermophilic organisms, *Methanothermobacter thermautotrophicus* and *Sulfolobus solfataricus* (54). At ambient temperature, both *Mth*MCM and *Sso*MCM form obligate homohexamers in solution with low DNA unwinding activity. We argue that the absence of a system that is experimentally tractable at lower temperatures has restricted characterization of the assembly of a homomeric MCM and thereby prevented a comparative analysis with heteromeric systems.

In the 25 years since archaeal MCMs were first characterized, new lineages of archaea have been discovered that inhabit diverse environments and with evermore closer evolutionary links to eukaryotes (55). We reasoned that characterizing the activity of MCMs across a broader range of archaeal organisms may uncover candidates that provide a better model for investigating the assembly of a homomeric MCM under ambient experimental conditions. Our current work identifies such a system—*Mac*MCM, the sole MCM from the ectosymbiotic archaea *M. acidophilum*. Experimental characterization of *Mac*MCM has allowed us to gain new insight into the assembly of a homomeric MCM onto a DNA substrate.

Evolution adapts organisms to their environments. At the protein level, enzymes have evolved to operate efficiently in conditions specific to the environmental niche of the organism. Under the ambient conditions of our activity screen, naturally occurring proteins from organisms that inhabit lower temperature environments showed the highest activity overall. Previous studies have demonstrated that *Mth*MCM and *Sso*MCM display robust activity at temperatures >50 °C (44, 47). However, in our assays, neither of these enzymes demonstrated substantial activity at 25 or 45 °C. The effect of temperature on MCM oligomeric state and morphology has been previously explored for these two MCMs. It was initially found that *Mth*MCM predominantly exists as a dodecamer at room temperature (27); however, when SEC analyses were later repeated at near-physiological temperatures (50 °C), *Mth*MCM populations redistributed into a hexameric species (30). Moreover, at a higher temperatures, a greater number of open ring-form hexamers of both *Mth*MCM and *Sso*MCM were observed by negative-stain EM (28, 31). *M. acidiphilum* is a mesophile, and thus, *Mac*MCM is likely adapted to operate in a lower temperature environment. Indeed, we observe that, unlike *Mth* or *Sso* proteins, *Mac*MCM is predominantly monomeric at room temperature.

Subunit–subunit interfaces of complexes from thermophilic organisms typically contain more hydrogen bonds and salt bridges than mesophilic counterparts: The interfaces observed in our *Mac*MCM crystal structure are more similar to yeast MCM in terms of polar noncovalent interactions than to an MCM from a thermophilic archaeon. At lower temperatures, higher numbers of intersubunit interactions may restrain conformational change within the hexamer and thereby reduce DNA unwinding activity. Indeed, structure-guided mutagenesis on both *Mth* and *Sso*MCM is consistent with these observations. For example, dodecamerization of *Mth*MCM can be inhibited by mutation of salt bridges that mediate head-to-head hexamer interactions (56, 57). Furthermore, removal of intersubunit salt bridges in the C-terminal domain of *Sso*MCM prevents hexamer formation of an unliganded enzyme (46). Reducing the number of intersubunit salt bridges in *Sso*MCM also resulted in an increase in helicase activity (46). It is plausible that monomeric species of *Sso*MCM and *Mth*MCM would be detectable if these systems could be studied under conditions closer to those of the environmental niche of these organisms. However, *Mac*MCM, our new model enzyme, provides convenient access to these states under ambient conditions. Furthermore, we were able to demonstrate that of the lower number of salt bridges that *Mac*MCM possesses, at least two are essential for proper hexamer assembly and that we were able to reinforce the stability of the oligomer with a limited set of rationally targeted mutations.

### Can *Mac*MCM form higher order oligomers?

The formation of an MCM double hexamer is thought to be an important step in replication initiation in both archaea and eukaryotes (27, 58). We saw no evidence that *Mac*MCM forms a double hexamer in either SEC or SEC–MALLS data under any of the conditions tested. Moreover, EMSAs only showed a single mobility shift across the concentration range tested, consistent with a single type of *Mac*MCM–DNA complex. However, the low intrinsic affinity for DNA in the absence of ATP does not preclude the formation of higher order complexes at elevated protein concentrations or with different classes of DNA substrate. *Sso*_N_*Pfu*_C_MCM, which bound forked DNA with much higher affinity than *Mac*MCM in our EMSA assays, did show evidence of forming two higher order oligomeric species in EMSAs, though it is unclear whether the larger species is a true double hexamer or simply two copies of a single hexamer bound to the same DNA. Notably, only a homohexamer was observed by SEC–MALLS analysis of *Sso*_N_*Pfu*_C_MCM at similar concentrations. While we do see crystal-induced asymmetry in the positioning of the six ZnFs, the structure and sequence of the NTD of *Mac*MCM appear compatible for the formation of a double hexamer, like those previously described for other archaea and eukaryotic MCMs.

### *Mac*MCM shows similar biochemical properties to core eukaryotic Mcm2–7

As with previously studied archaeal MCMs, *Mac*MCM can unwind forked DNA *in vitro*. However, real-time analysis shows multiphase unwinding kinetics (Fig. 4*A*) that resembles the *in vitro* properties of eukaryotic Mcm2–7. As part of the CMG complex, Mcm2–7 also exhibits sigmoidal unwinding kinetics, with the maximal unwinding rate that achieved ∼5 to 10 min after addition of ATP (41). A similar scale delay was reported for the interaction of Mcm2–7 with ssDNA in the presence of ATP, indicating that this “slow” kinetic property is intrinsic to the core Mcm2–7 subunits and independent from protein cofactors (20). Structures of Mcm2–7 without DNA reveal that the six core subunits form a spiral-like arrangement (59), whereas in the CMG complex, the subunits adopt a planar configuration in which Mcm2 and Mcm5 are stabilized by Cdc45 and GINS (39). In both structures, the WHD of Mcm5 occupies a position in the central channel and must presumably be displaced for DNA unwinding to occur (39, 40). In our experiments, truncation of the WHD of *MacM*CM eliminated multiphase kinetics, at least at the resolution of the assay. To our knowledge, equivalent experiments with a truncated Mcm5 that lacks the WHD have not been performed.

### The affinity of *Mac*MCM for DNA is impacted by the presence of nucleotides and the WHD

While the importance of the interplay of DNA, ATP, and WHDs in the assembly of Mcm2–7 is now well established, our study is the first to be able to explore this interplay in a homohexameric MCM complex. *Mac*MCM binds to DNA less strongly than the chimeric *Sso*_N_*Pfu*_C_MCM; however, ATP has a stronger effect on the affinity of *Mac*MCM for DNA compared with *Sso*_N_*Pfu*_C_MCM. More specifically, constructs of *Mac*MCM that can hydrolyze ATP show the highest affinity for DNA. However, consistent with studies that have used AMP–PNP to explore loading of Mcm2–7 onto DNA in the absence of hydrolysis (41), ATP turnover is not strictly required for *Mac*MCM DNA loading, as the inactive variant *Mac*MCM^E391Q^ coelutes as a hexamer with DNA in-gel filtration. Moreover, in the presence of ATP, *Mac*MCM constructs that lack the WHD interact with forked DNA more strongly than full-length equivalents. Therefore, binding and catalysis of ATP and displacement of the WHD are conserved steps in the assembly of MCM subunits onto DNA.

In summary, we report the first assessment of the assembly pathway of an archaeal MCM onto a DNA substrate. This work was made possible *via* the discovery and characterization of an experimentally tractable MCM from a mesophilic archaeon. All extant MCMs have evolved from a homohexameric ancestor, but the choice of archaeal subjects used in previous structure–function studies has inadvertently limited our capacity to probe the differences and similarities that exist between eukaryotic and archaeal MCMs. The data we present here reveal that the interplay of DNA, ATP, and WHDs in the assembly pathway is conserved between homomeric and heteromeric MCMs and that these properties are intrinsic features of the basic MCM subunit. The fact that such similarities exist suggests that the fundamental steps of MCM assembly evolved before the appearance of additional regulatory factors.

## Experimental procedures

### Preparation of recombinant MCM samples

MCM genes were synthesized and cloned into the ampicillin-resistant pONT vector by GenScript (Table S1). Genes were positioned downstream of a T7 promoter and N-terminally His-10 tagged. All genes were codon optimized for expression in *E. coli*, and a double stop codon was added. Where performed, site-directed mutagenesis was carried out using QuikChange Lightning Mutagenesis (Agilent), according to the manufacturer's guidelines. MCM protein was overproduced in *E. coli* BL21 (DE3) pLysS cells (Agilent). Cultures were grown at 37 °C in LB containing 34 μg/ml chloramphenicol, 100 μg/ml ampicillin, and 1% (w/v) glucose. When cultures reached an absorbance of 0.6 to 0.8 at 600 nm, expression was induced with 1 mM IPTG and placed at 20 °C. After 20 h, the final absorbance at 600 nm was measured, and cells were harvested *via* centrifugation at 4000*g*. Cell pellets were then stored at −80 °C until required.

### Purification of recombinant MCMs

Cell pellets (from 200 ml culture) were thawed and resuspended in buffer A (20 mM Tris–HCl [pH 8.0], 500 mM NaCl, 20 mM imidazole, and 5% [w/v] glycerol) to an absorbance at 600 nm of 100. Buffer A was supplemented with DNase, RNase (both at 20 μg/ml), and cOmplete protease inhibitor tablets (Roche). Cells were lysed by sonication at 70 W (3 s on, 7 s off for 1 min per 100 ml culture). Cell extract was then centrifuged for 45 min at 30,000*g*, 4 °C. The resulting supernatant was loaded onto a 1 ml HisTrap FF column (GE Healthcare) and pre-equilibrated in buffer A. The column was then washed with a high-concentration salt wash (20 mM Tris–HCl [pH 8.0], 2 M NaCl, 20 mM imidazole, and 5% [w/v] glycerol) before being re-equilibrated into buffer A. Bound protein was eluted from the column with buffer B (20 mM Tris–HCl [pH 8.0], 500 mM NaCl, 500 mM imidazole, and 5% glycerol). At this point, if samples were being used in the initial characterization screen, fractions were pooled and dialyzed against buffer C (20 mM Tris–HCl [pH 8.0], 500 mM NaCl, and 5% [w/v] glycerol) overnight at 4 °C. Dialyzed protein was spin-concentrated in a Vivaspin 6 molecular weight cutoff 50,000 (Sartorius) to the desired concentration, then snap frozen in aliquots, and stored at −80 °C. For all other samples, positive fractions from elution B were pooled, and tobacco etch virus protease was added to a ratio of 1 mg tobacco etch virus:50 mg His-tagged protein. Fractions were then dialyzed against buffer D (20 mM Tris–HCl [pH 8.0], 500 mM NaCl, 1 mM DTT, and 5% glycerol) overnight at 4 °C. Tag cleavage was confirmed by SDS-PAGE. Dialysate was then loaded onto a HisTrap FF column, and the recombinant protein was collected from the flow through. The flow through was then concentrated in an Amicon Ultra-15 50,000 molecular weight cutoff spin concentrator to ∼10 to 20 mg/ml and loaded onto a HiPrep 26/60 S200 Size-Exclusion Column (GE Healthcare) and equilibrated in buffer D. Fractions were collected and spin concentrated as before to a final concentration of ∼7 to 20 mg/ml. Samples were either snap frozen in liquid nitrogen and stored at −80 °C or used immediately to set up protein crystallization experiments.

### Fluorescent helicase assay

Helicase unwinding reactions were carried out on a forked DNA substrate, which was formed by annealing a 5′-Cy3-labeled oligonucleotide 5′-[Cy3]GGGACGCGTCGGCCTGGCACGTCGGCCGCTGCGGCCAGGCACCCGATGGC(GTTT)_6_-3′; Merck) to a 3′-BHQ2-labeled oligonucleotide (5′-(TTTG)_8_CCGACGTGCCAGGCCGACGCGTCCC[BHQ2]-3′; Eurofins). A scavenger oligonucleotide (5′-GGGACGCGTCGGCCTGGC-3′; Merck) complementary to the duplex region of the BHQ2-labeled strand was added to the reaction in 10-fold excess to prevent reannealing of the unwound substrate. Standard reactions containing 1000 nM helicase (based on hexamer MW), 50 nM forked DNA, and 500 nM scavenger were monitored at 25 °C for 30 min with a sampling frequency of one reading per well per minute. Unless stated, the reaction buffer contained 250 mM potassium glutamate, 20 mM potassium phosphate (pH 8.0), 1% glycerol, 4 mM ATP, and 10 mM MgCl_2_.

### Crystallization and data collection

Samples of *Mac*MCM^ΔWHD.E391Q^ were dialyzed overnight at room temperature into 100 mM NaCl, 20 mM Tris (pH 8.0), 0.5 mM Tris(2-carboxyethyl)phosphine, and 5% glycerol. ATP (10 mM) and MgCl_2_ (10 mM) were added 10 min before setting up the crystallization condition. Long plate–shaped crystals grew over 3 days at 20 °C in a sitting drop containing 10 μl of protein solution and 10 μl of well solution (0.03 M NPS, 0.1 M Mops/Hepes [pH 7.5], 10% w/v PEG 20,000, and 20% v/v PEG MME 550). Crystals were harvested using a cryo-loop (Crystal Cap HP) and flash frozen in liquid nitrogen. Data were collected at Diamond Lightsource Beamline i03 at a wavelength of 0.976 Å and temperature of 100 K. Data were scaled and integrated using Xia2-DIALS software package to 2.59 Å resolution (60).

### Structure solution and refinement

Initial phases were calculated using Phaser molecular replacement software (61), which placed six copies of a no loop, polyalanine model of the AAA+ domain of *Sso*_N_*Pfu*_C_MCM in a ring (PDB code: 4R7Y) (35)). The R-free test set was generated by phenix.refine, which is set to pick 5% or at most 2000 reflections. Following the placement of this model, the electron density map was improved using RESOLVE density modification (60). Loops and the entire NTD were then built iteratively using manual building in Coot and the automated software AutoBuild and Buccaneer (62, 63, 64). Refinement was carried out using phenix.refine (65)). Following the building of the NTD, zinc ions were placed through observation of weak anomalous data. Anomalous data were also checked to rule out coordination by the OB-folds of sulfur groups present in the buffer (*e.g.*, Mops or sulfate) instead of phosphate. Cysteine co-ordination and ligand restraints were then generated using ReadySet! (66). Where present, nucleotide in the active sites was modeled as ADP.

### Analytical SEC

A Superose 6 Increase 10/300 GL or Superdex S200 Increase 10/300 GL (where stated) was pre-equilibrated with 200 mM NaCl, 20 mM Tris (pH 8.0), and 5% glycerol on an ÄKTA Pure (GE Life Sciences). Where nucleotide was present, the running buffer also included 1 mM ATP and 10 mM MgCl_2_. MCM was diluted to 60 μM monomer concentration in running buffer. Where ligands were present, 5 mM ATP, 10 mM MgCl_2_, or 10 μM DNA was added to the sample. Samples (100 μl) were loaded and run at 0.5 ml/min. Absorbance was simultaneously measured at 290 and 495 nm. The column was calibrated using thyroglobulin (660 kDa), β-amylase (223 kDa), alcohol dehydrogenase (150 kDa), carbonic anhydrase (30 kDa), and cytochrome *C* (12 kDa; all from Merck). The relationship between elution volume and molecular mass was determined using linear regression. Fluorescein-labeled ssDNA substrate (5’[FAM]-pT_50_; Merck) was used in all SEC experiments to allow us to characterize complexes formed on DNA in the absence of any unwinding.

### Size-exclusion chromatography with multiangle laser light scattering

A Superose S6 Increase 10/300 GL analytical column (GE Healthcare) was equilibrated overnight with 200 mM KCl, 50 mM Tris–Cl (pH 8.0), 5% (v/v) glycerol, and 0.5 mM DTT buffer on a Shimadzu HPLC system. A total of 100 μl protein at 1 to 10 mg/ml was passed over the column at a flow rate of 0.5 ml/min. Light scattering was determined using a Wyatt HELEOS-II MALLS detector. Differential refractive index was determined using a Wyatt rEX refractive index detector. Data were analyzed using Astra 7 (Wyatt) software, where the MW is calculated from a Zimm model. Bovine serum albumin run at 2.5 mg/ml was used to normalize the differential refractive index signal. The *dn/dc* value was adjusted until the expected MW of bovine serum albumin (66 kDa) was obtained.

### Fluorescent anisotropy

Helicase and DNA mixes were prepared in 250 mM potassium glutamate, 20 mM Tris (pH 8.0). A forked DNA substrate was prepared by annealing a 5ʹ-FAM-labeled oligonucleotide (5ʹ-[FAM]GGGACGCGTCGGCCTGGCACGTCGGCCGCTGCGGCCAGGCACCCGATGGC(GTTT)_6_-3ʹ; Merck), with a partially complementary oligonucleotide (5ʹ-(TTTG)_8_CCGACGTGCCAGGCCGACGCGTCCC-3ʹ; Merck). Forked DNA was added to one in two serially diluted MCM samples, with each well containing a final concentration of 1 nM DNA. Where present, binding reactions were supplemented with 4 mM nucleotide and 10 mM MgCl_2_. The average change in anisotropy (ΔA) across three technical repeats was calculated and fitted to a Langmuir binding isotherm:

$$\Delta A=\frac{{\Delta A}_{\max}\times {\left[MCM\right]}^{n}}{{\left[MCM\right]}^{n}+{K_{d}}^{n}}$$

where Δ*A*_max_ is the maximum change in anisotropy, *K*_*d*_ is the equilibrium dissociation constant, and *n* is a Hill coefficient. The presence of a Hill coefficient is justified by the observation of multiple MCM binding stoichiometries in EMSA. Model fitting was performed using a nonlinear least squares function in R.

### Electrophoretic mobility shift assays

Agarose gels (0.8% [w/v]) were prepared in 1x Tris–borate (90 mM Tris, 90 mM borate, pH 8.3) buffer. Protein and buffer were prepared in 250 mM KGlu and 20 mM Tris–Cl (pH 8.0). A forked DNA substrate was prepared by annealing a 5ʹ-FAM-labeled oligonucleotide (5ʹ-[FAM]GGGACGCGTCGGCCTGGCACGTCGGCCGCTGCGGCCAGGCACCCGATGGC(GTTT)_6_-3ʹ; Merck), with a partially complementary oligonucleotide (5ʹ-(TTTG)_8_CCGACGTGCCAGGCCGACGCGTCCC-3ʹ; Merck). Forked DNA was added to one in two serially diluted MCM samples, with each sample containing a final concentration of 10 nM DNA. A DNA-only control was also prepared to determine the motility of unbound DNA. Samples were then incubated at room temperature for 30 min. Before loading, 20 μl of 2x Tris–borate and 25% (v/v) glycerol were added to each sample. Each sample (10 μl) was then run on the agarose gel for 20 min at 150 V. Gels were imaged on a Typhoon scanner (GE Healthcare) using Cy2 filters with a 100 μm imaging pixel size. To estimate the equilibrium dissociation constant (*K*_*d*_) between protein and DNA, the protein concentration is identified where the DNA motility is distributed equally between free and protein-bound states.

### NMR spectroscopy

NMR spectroscopy was used to confirm ATPase activity of purified MCM samples. Each reaction mixture (600 μl) contained 50 mM ATP, 2.5 Mm MgCl_2_, 10% (v/v) D_2_O, 250 Mm KGlu, and 10 mM Tris–Cl (pH 8.0). Reactions were initiated by adding MCM to a final concentration of 50 μM (monomeric). After 30 min at 25 °C, 1D [^31^P]-NMR spectra were recorded on a 500 MHz NMR spectrometer (Bruker). Spectra were processed using TopSpin software (Bruker).

## Data availability

The atomic model described in this study and accompanying structure factors have been deposited to the PDB under the accession code 8Q67.

## Supporting information

This article contains supporting information (27, 29, 44, 50).

## Conflict of interest

The authors declare that they have no conflicts of interest with the contents of this article.
