# Supplementary material for: Assembly of a homohexameric minichromosome maintenance complex is dependent on ATP and DNA
Source: J Biol Chem. 2025 Dec 17;302(4):111072. doi: 10.1016/j.jbc.2025.111072 (PMC13049513; doi:10.1016/j.jbc.2025.111072)
Supplement: Supplementary Material 1 [file mmc1.pdf]

Assembly of a homohexameric minichromosome maintenance complex is dependent on ATP and DNA

Oliver W Noble[1], Clement Degut[1], Michael R Hodgkinson[1], James P J Chong[1,2,*], Michael J Plevin[1,3,4,*]

Supplementary Material

Supplementary Figures 1-18

Supplementary Tables 1-3

Supplementary Table 1: The optimal growth temperatures of archaeal species targeted in this study.

* Optimal growth temperature data is limited for the organism. The conditions of the habitat where the organism was initially identified is taken as a proxy for the organisms potential growth range.

† Full gene contains a highly conserved internal intein that is removed in our construct.

					Temperature (ºC)				
Organism	MCM	UniProt Accession Code	N_{res}	MW	Min	Optimum	Max	Phyla	Reference
Archaeoglobus fulgidus	*Afu*MCM	Q7ZAA5	698	78,784	60	83	85	Euryarchaeota	[1]
Aeropyrum pernix	*Ape*MCM	Q9YFR1	697	78,472	70	90	97	TACK	[2]
Haloferax volcanii	*Hvo*MCM	D4GZG5	702	78,855	30	42	55	Euryarchaeota	[3]
Korarchaeum cryptofilum	*Kcr*MCM	A2BL91	703	79,373	55	85	90	TACK	[4]
Mancarchaeum acidophilum	*Mac*MCM	A0A218NN99	687	76,126	10	37	45	DPANN	[5]
Methanosarcina barkeri	*Mba*MCM	A0A0E3QYF9	700	78,909	30	35	45	Euryarchaeota	[6]
Methanohalophilus halophilus	*Mha*MCM	A0A1L3PZK3	696	78,154	30	40	55	Euryarchaeota	[7]
Methanopyrus kandleri	*Mka*MCM	Q8TWR7	656	74,062	84	98	110	Euryarchaeota	[8]
Methanothermobacter thermautotrophicus	*Mth*MCM	O27798	666	75,553	40	65	75	Euryarchaeota	[9]
Nanohaloarchaea archaeon SG9	*Nac*MCM	A0A1D8MR82	675	76,073	16	33	40	DPANN	[*]
Nanoarchaeum equitans	*Neq*MCM	Q74MT7	657	74,097	75	80	95	DPANN	[10]
Nitrosopumilus maritimus	*Nma*MCM	A9A310	695	78,136	15	28	32	TACK	[11]
Pyrococcus furiosus	*Pfu*MCM	Q8U3I4 †	681	76,704	70	95	103	Euryarchaeota	[12]
Saccharolobus solfataricus	*Sso*MCM	Q9UXG1	686	77,428	55	75	90	TACK	[13]

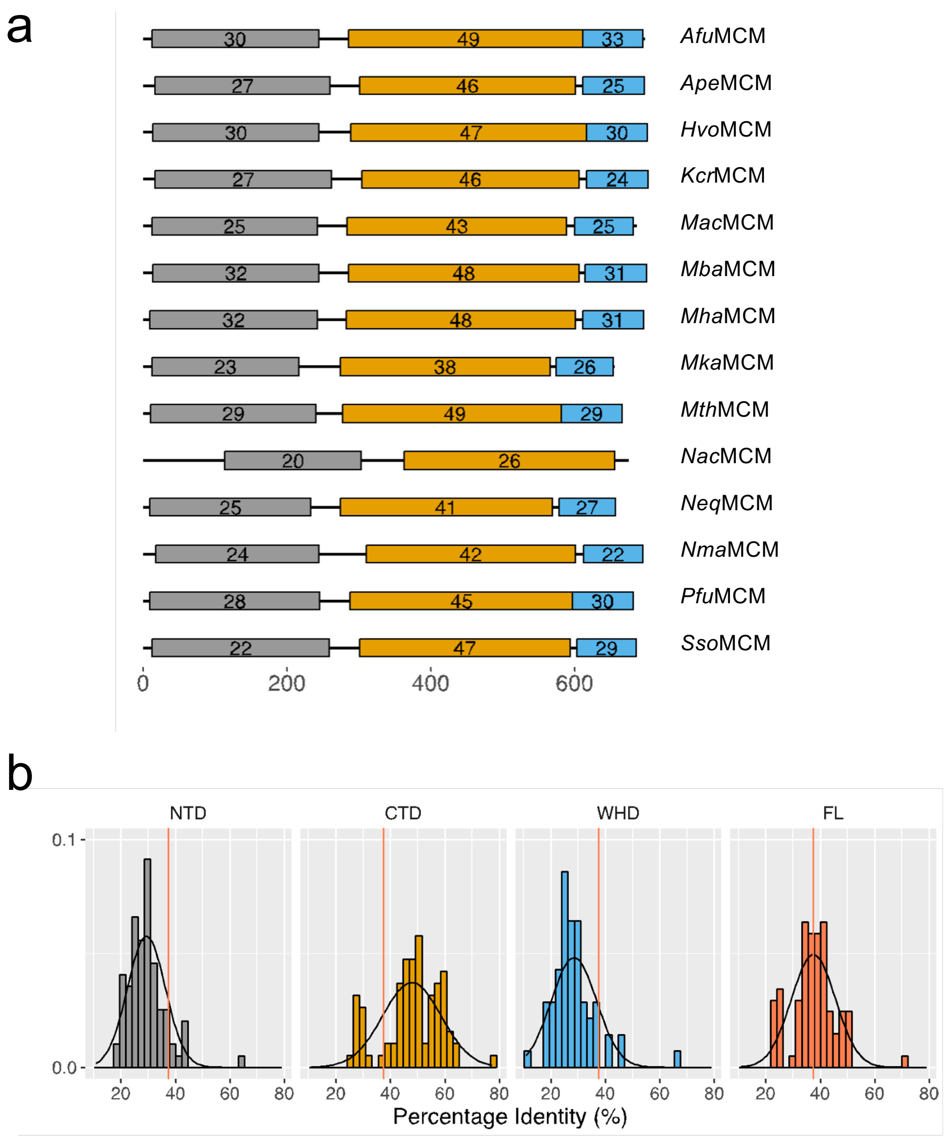

Supplementary Figure 1: Schematic and conservation analysis of MCM used in this study. **a** Conserved MCM subdomains domains were identified using the InterProScan[14] tool (https://www.ebi.ac.uk/interpro/search/sequence/). Coloured boxes indicate the identified domain and length (amino acids), where grey is a N-terminal DNA binding domain, orange is a P-type NTPase and blue is a winged-helix domain (WHD). Numbers inside the box indicate the average percentage identity score for each MCM subdomain when aligned to all MCMs studied here (https://www.ebi.ac.uk/Tools/msa/clustalo/). **b** The distribution of percentage identity scores for all MCM and subdomains. The black line represents the normal distribution, with a standard deviation σ and mean μ. The red line represents the mean value for the full-length enzyme. **NTD**: μ=29.4, σ=6.9; **CTD**: μ=47.9, σ=10.7; **WHD**: μ=28.5, σ=8.3; **FL**: μ=37.5, σ=8.1).

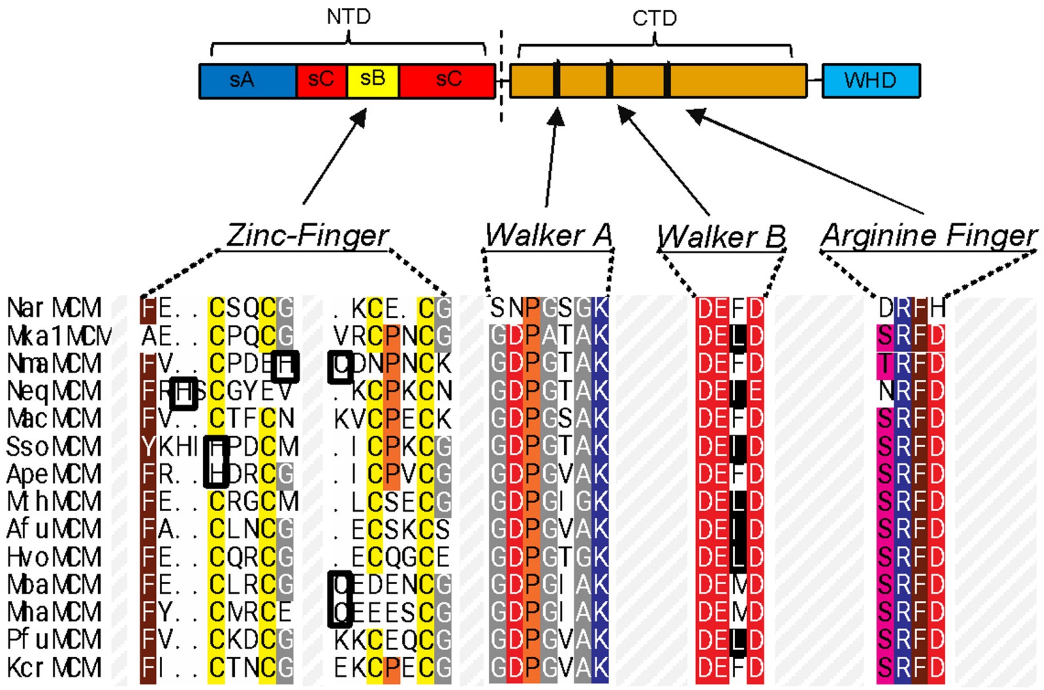

Supplementary Figure 2: Sequence alignment of core MCM motifs. MSA of MCM used in this study. Sequences were aligned using Clustal Omega[15] and visualized using the TexShade package in LaTeX. Conserved amino acids are coloured based on the chemical properties of the functional side chain group. The conserved glutamate (E) residue in the Walker B motif is mutated to glutamine (Q) for *Mac*MCM^{E391Q}. Brown: aromatic, Yellow: sulphur, Orange: imino, Grey: aliphatic (small), Red: acidic, Blue: basic, Black: aliphatic, Purple: hydroxyl. Black boxes show alternative residues that are predicted to ligate the Zinc in the Zinc-finger domain.

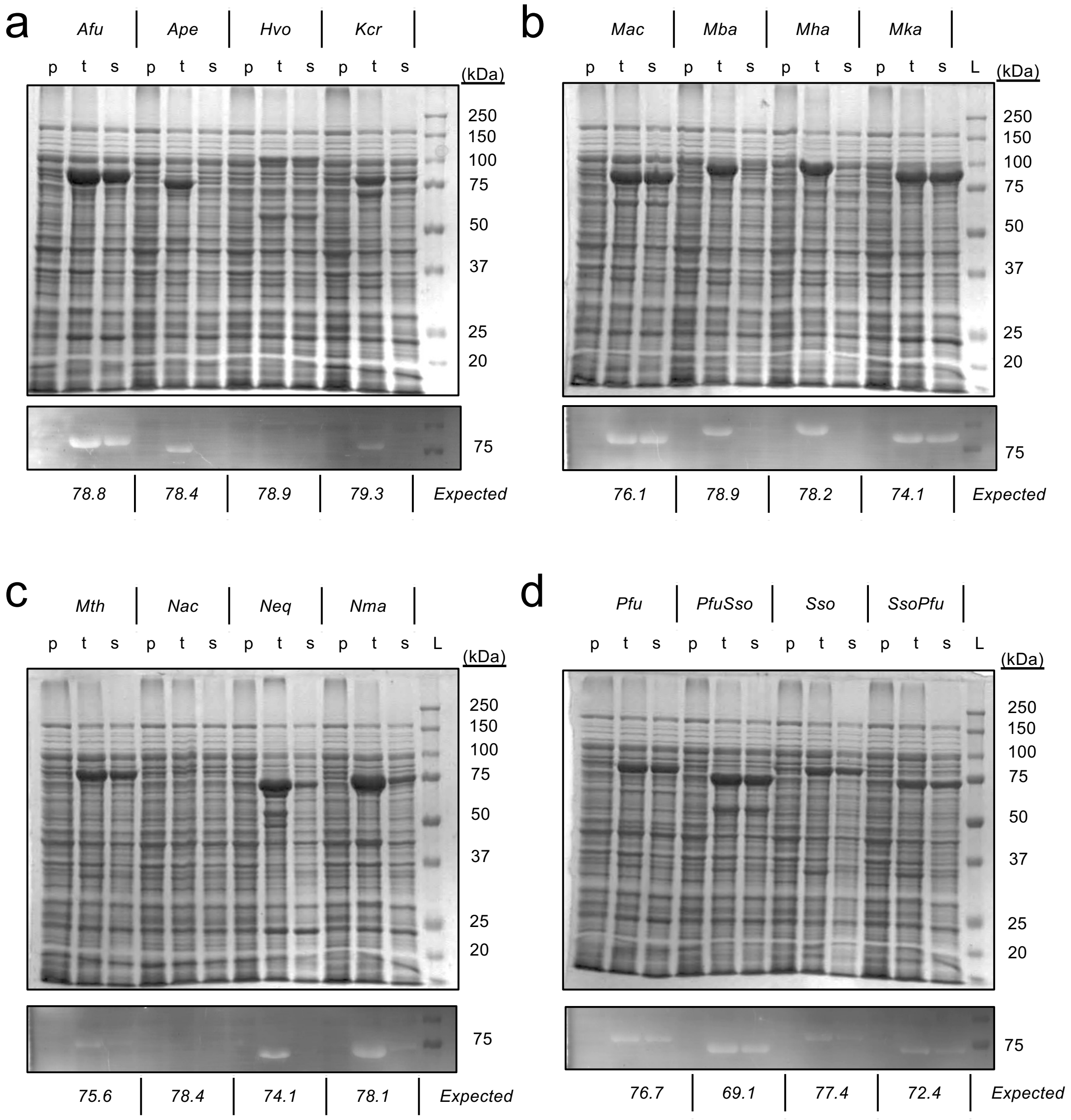

Supplementary Figure 3: Test for expression and solubility of 16 archaeal MCM orthologues. a-d All samples were analysed by SDS-PAGE using on a 12 % acrylamide gels. Samples represent pre-induction of expression (p), then total (t) and soluble (s) fractions which are collected 20-hours after the IPTG- induction at 20 °C. Top panels represent gels where proteins are stained through a Coomassie- based approach. Bottom panels represent gels where proteins are stained using a fluorescent Ni-NTA conjugate that indicates the presence of poly-histidine tags. Reference (L) lanes represent MW standard marker (Precision Plus Protein™ All Blue Pre-Stained Protein Standards).

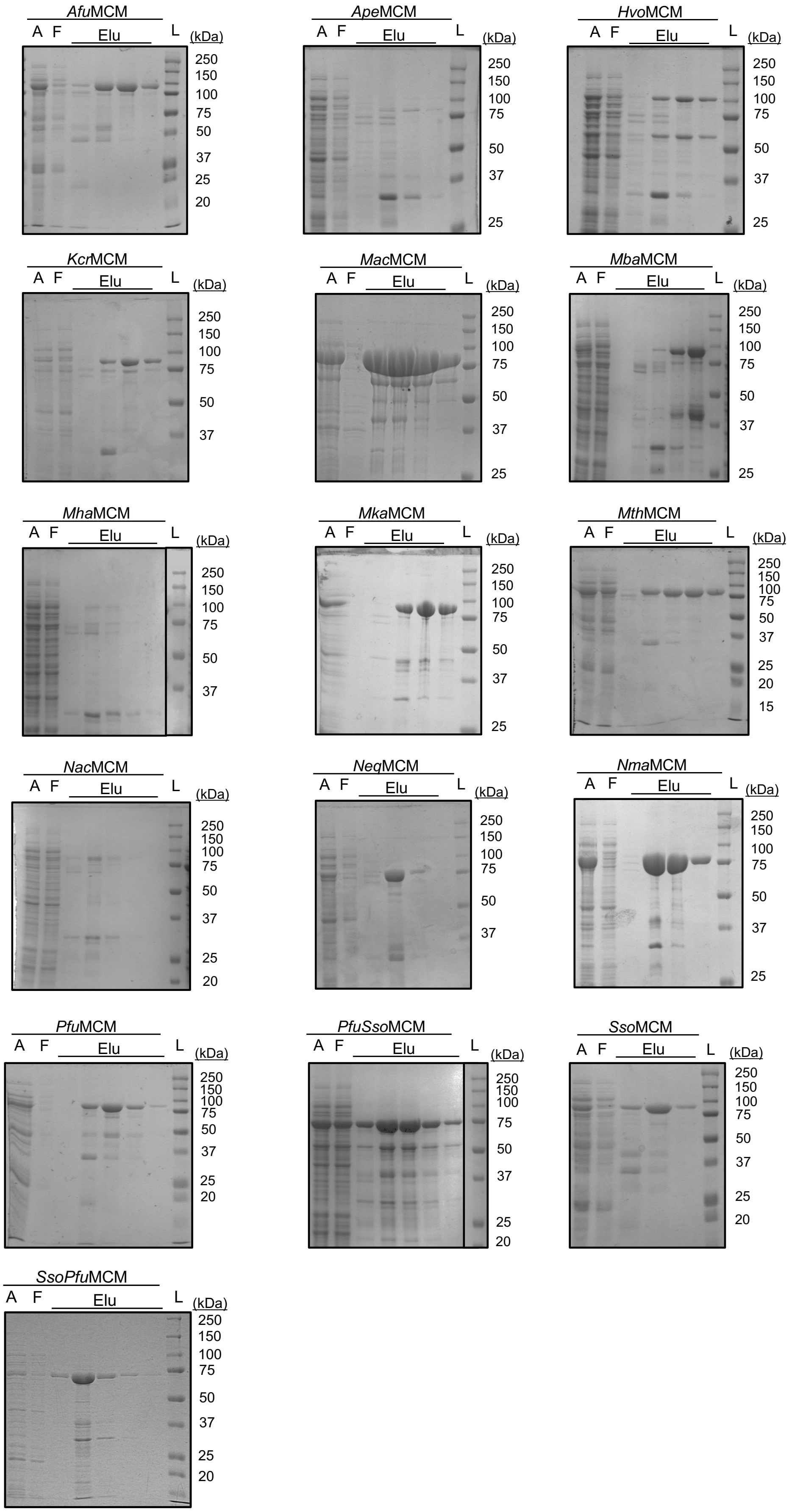

Supplementary Figure 4: Purification of His_{10}-labelled recombinant MCM. Fractions from IMAC purification were analyzed by SDS-PAGE on 12 % (*w/v*) polyacrylamide gels. Fractions include the sample applied to the Ni-NTA column (A), the column flow through (F) and select elutions (Elu). Reference (L) lanes represent MW standard marker (Precision Plus Protein™ All Blue Pre-Stained Protein Standards).

Supplementary Table 2: Estimated protein concentration and levels of DNA contamination following one-step IMAC purification.

Identifier	Raw Yield (mg per 200 mL culture)	$A_{260/280}$
*Afu*MCM	4.1	0.83
*Ape*MCM	1.0	1.01
*Hvo*MCM	1.2	1.03
*Kcr*MCM	3.5	0.95
*Mba*MCM	1.2	0.92
*Mha*MCM	0.1	0.87
*Mac*MCM	18.0	0.63
*Mka*MCM	3.2	0.76
*Mth*MCM	5.7	0.82
*Nac*MCM	0.1	1.24
*Neq*MCM	1.5	0.76
*Nma*MCM	3.4	0.86
*Pfu*MCM	2.3	0.82
$Pfu_N Sso_C$MCM	2.4	0.71
$Sso_N Pfu_C$MCM	2.3	0.63
*Sso*MCM	2.8	0.88

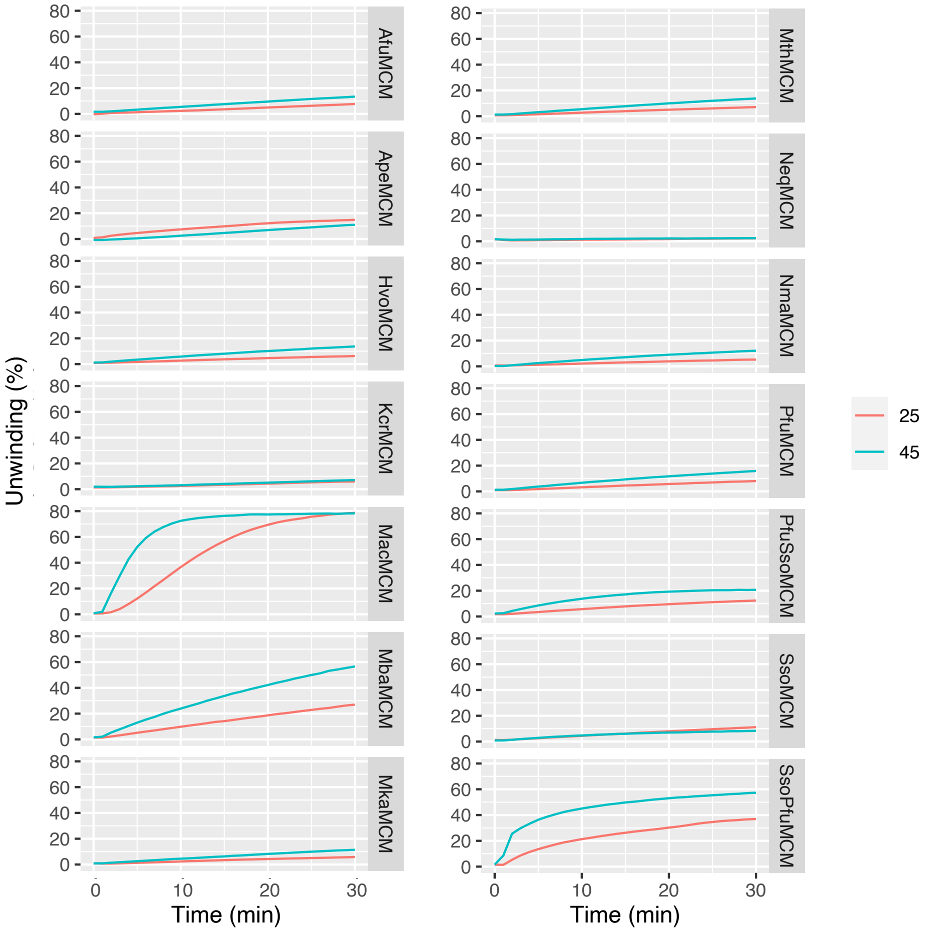

Supplementary Figure 5: Real time unwinding traces for crude screens. Real time DNA-unwinding curves for the stated MCMs measured at 25 °C (orange) and 45 ºC (cyan). The slight sigmoidal shape seen for *SsoPfu*MCM was not evident when a pure sample was assays (see Supplementary Figure 7)

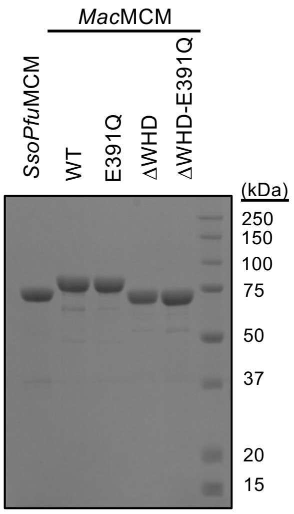

Supplementary Figure 6: Purification of recombinant MCM. Purity analysis of recombinant proteins. SDS-PAGE analysis of 3 μg each purified MCM. WT, Wild-type; WHD, winged-helix domain.

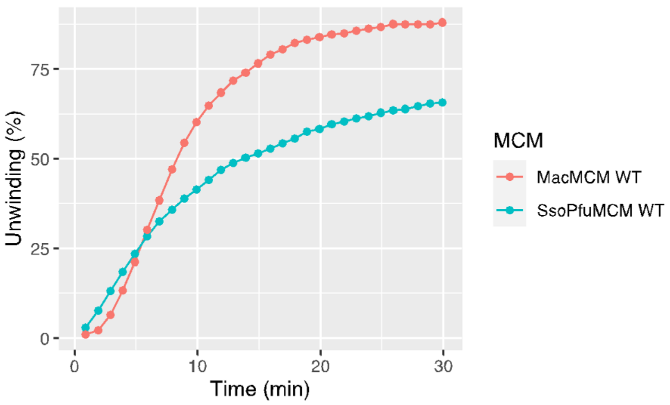

Supplementary Figure 7: DNA unwinding kinetics of pure *SsoPfu*MCM. Real time DNA-unwinding curves for purified samples of *Mac*MCM and *SsoPfu*MCM at 25 °C.

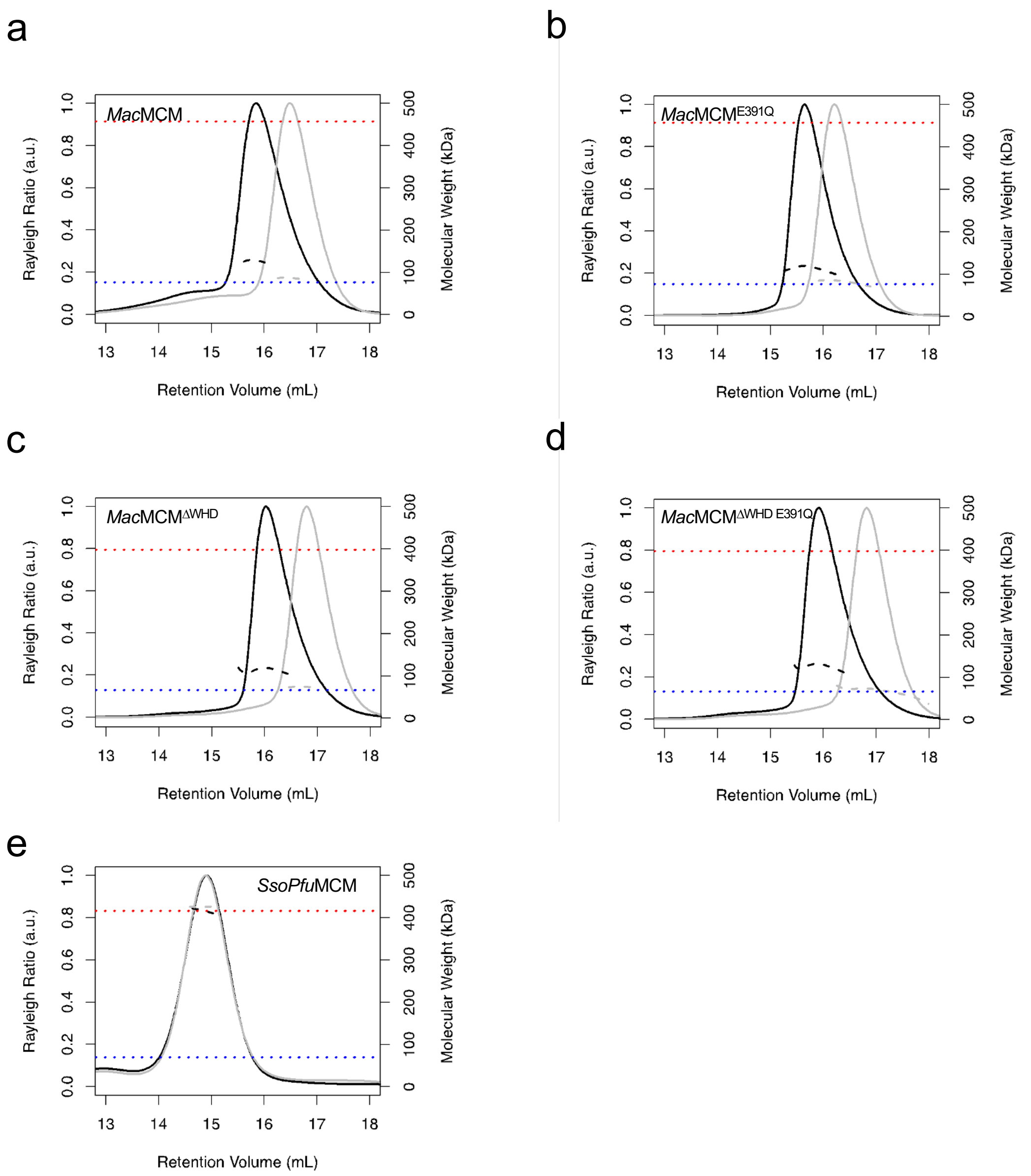

Supplementary Figure 8. Oligomeric state of MCM samples in the absence of ligands.
a-e SEC MALLS elution profile of the stated MCM at concentrations of 1 mg/mL (grey; ~2 μM) or 10 mg/mL (black; ~20 μM) from a Superose 6 Increase column was monitored through light scattering (Rayleigh ratio). The Rayleigh ratio was normalised to the height of the main peak. Calculated molar mass values are shown as a dotted line. For each sample, expected molar masses are shown for a monomer (blue) and a hexamer (red).

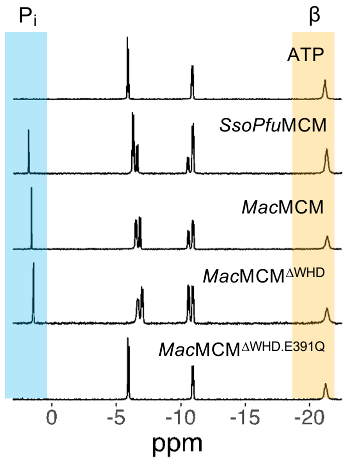

Supplementary Figure 9: ^{31}P-NMR confirms inactivity of ATPase mutants. The presence of inorganic phosphate was determined for the stated MCM sample after 30 minutes using ^{31}P-NMR. The top panel represents 50 mM ATP sample, without MCM. In each experiment, a final concentration of 8.3 μM MCM was added to ATP. The blue region highlights the expected region for the inorganic phosphate peak (P_i), whilst the orange region highlights the expected region for the β-phosphate of ATP.

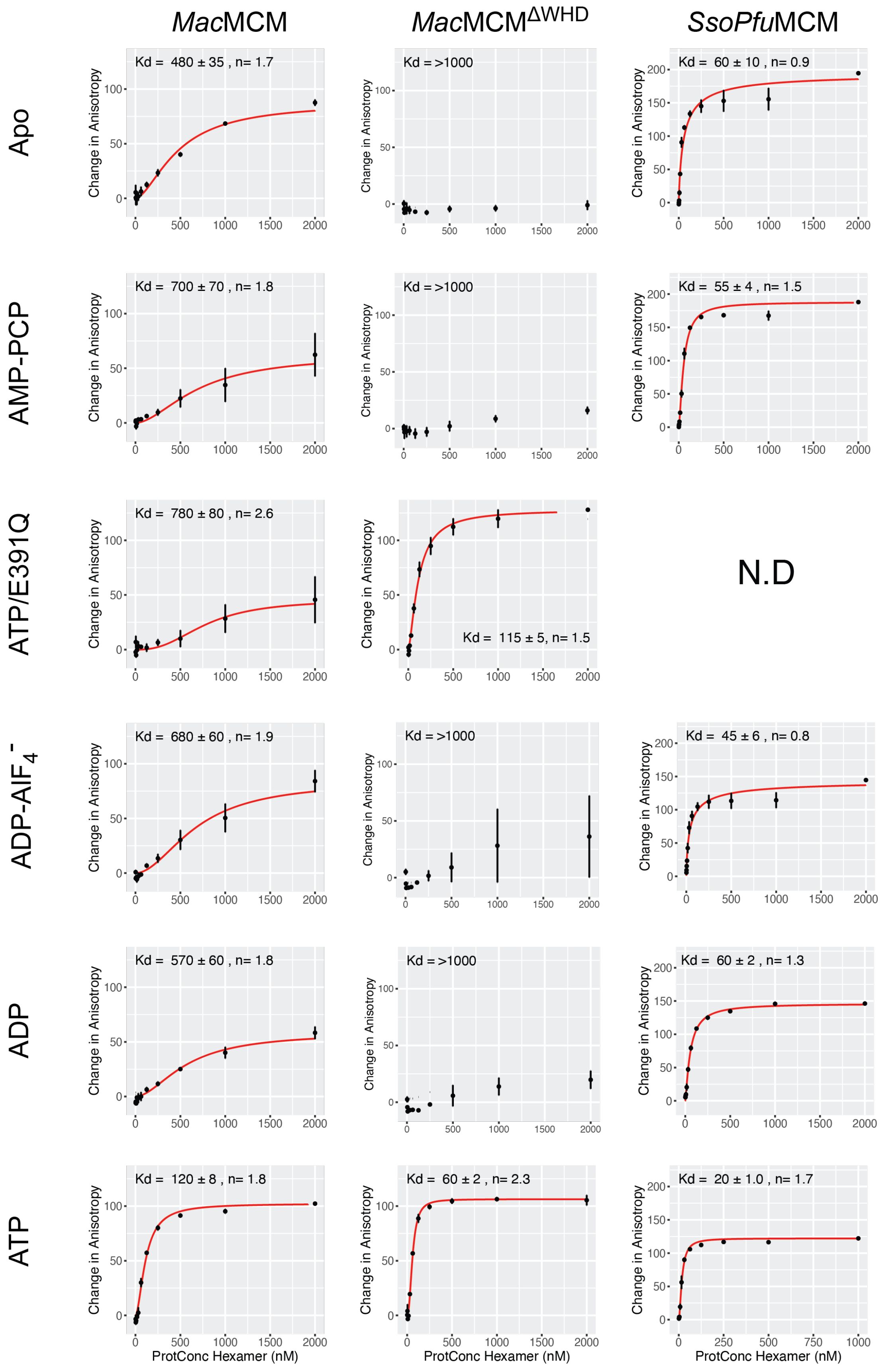

Supplementary Figure 10. Assessment of MCM-DNA interactions in response to different nucleotides. The binding of MCM to a forked DNA substrate was measured by fluorescence anisotropy. MCM were mixed at the stated concentration (nM hexamer) with 1 nM FAM-labelled forked DNA substrate and incubated for 30 minutes at 25 °C. Measurements were performed on a Clariostar plate reader (BMG Labtech). Anisotropy values were standardized by subtraction of a no-protein control well and fitted to Langmuir binding isotherm with Hill coefficient. Error bars represent +/-1 standard error of the mean, where n=3.

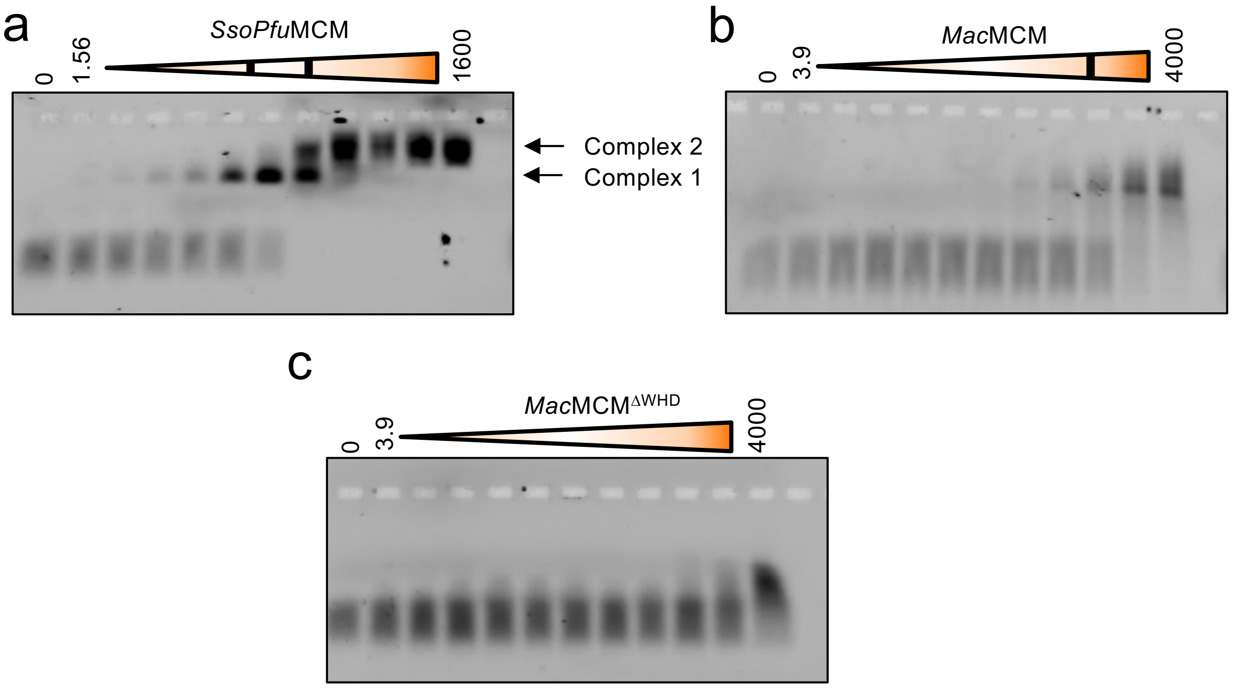

Supplementary Figure 11. Assessment of DNA binding by EMSA. a-c MCM DNA binding was measured by EMSA. 10 nM fluorescein-labelled forked DNA substrate was mixed with different concentrations of each MCM and incubated for 30 minutes at 25 ºC Samples were resolved on a 1 x TB 0.8 % agarose gel and imaged using a Typhoon gel scanner. Horizontal triangles indicate maximum and minimum protein concentrations used in titrations with intermediate concentrations produced by 1:2 serial dilution.

**Supplementary Table 3<br>Crystallographic statistics**	
**Data collection**	
Space group	C 1 2 1
Cell dimensions	
a,b,c (Å)	228.70 127.49 177.04
α, β, γ (˚)	90.00 91.71 90.00
R_{meas}	0.115 (4.564)
I/σ <I>	11.3 (0.3)
Completeness (%)	99.99 (98.2)
Multiplicity	6.8 (7.0)
$CC_{1/2}$	1.0 (0.5)
**Refinement**	
Resolution (Å)	2.59 - 57.15 (2.59 – 2.64)
Unique reflections	157088 (15547)
R_{work}/R_{free}	0.231 / 0.253
No. of atoms	
Macromolecules	26931
Ligands	176
Solvent	13
B-factor (Å^2)	
Macromolecules	96.43
Ligands	101.79
Solvent	67.64
R.m.s deviations	
Bond lengths (Å)	0.018
Bond angles (º)	1.49

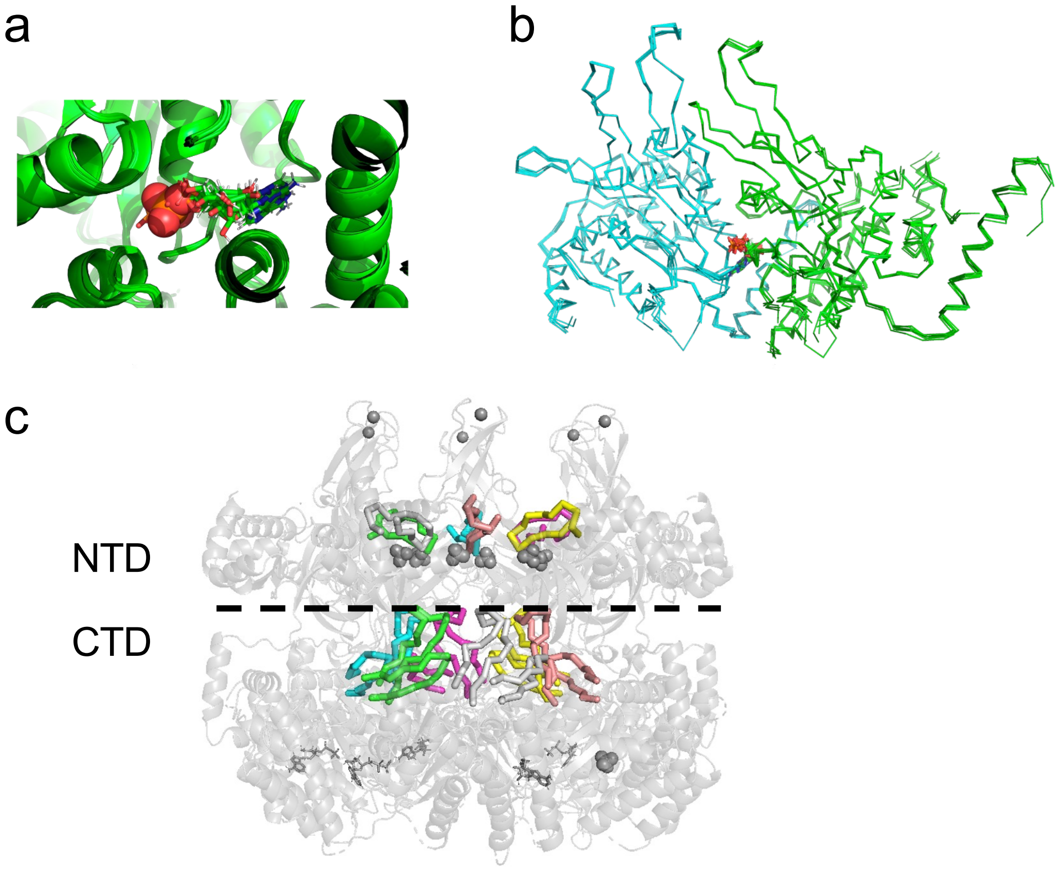

Supplementary Figure 12: C-terminal domains are structurally indistinct. a View of structurally aligned ATPase active sites. Protein is represented in cartoon format. Ligands are represented in sphere (phosphate) and stick (ADP) formats. The phosphate is highlighted to emphasise that it mimics the position of the beta-phosphate of ADP. **b** View of structurally aligned ATPase active site pairs, visualized in ribbon format. This *cis*-acting subunit is coloured in cyan, whilst the *trans*-acting subunit is coloured in green. **c** Positioning of DNA-binding hairpins with respect to the N and C-terminal tiers of the *Mac*MCM hexamer (grey). Hairpins are displayed in ribbon format and coloured by subunit.

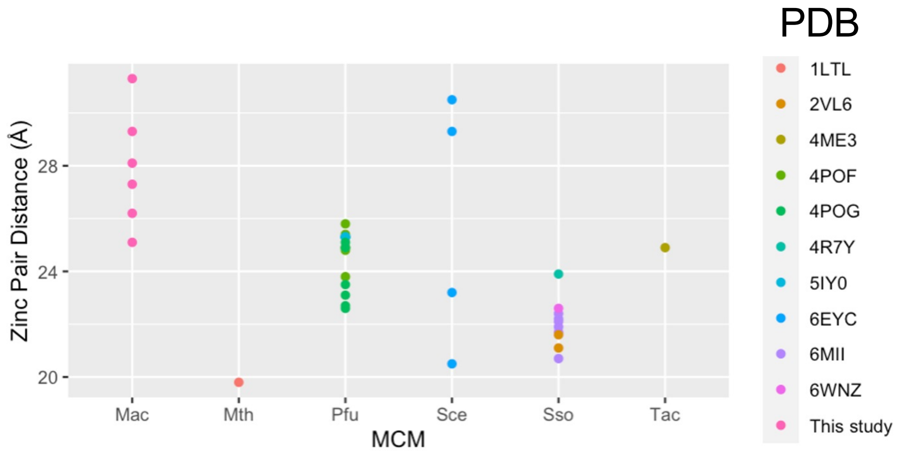

Supplementary Figure 13: The distance between neighbouring Zinc fingers in *Mac*MCM.
The distance between neighbouring *Mac*MCM Zinc Fingers was compared between structures published for archaeal and eukaryotic MCMs. Distances measured pertain to the position of the zinc-ion relative to the position of the zinc-ion on the neighbouring subunit. .

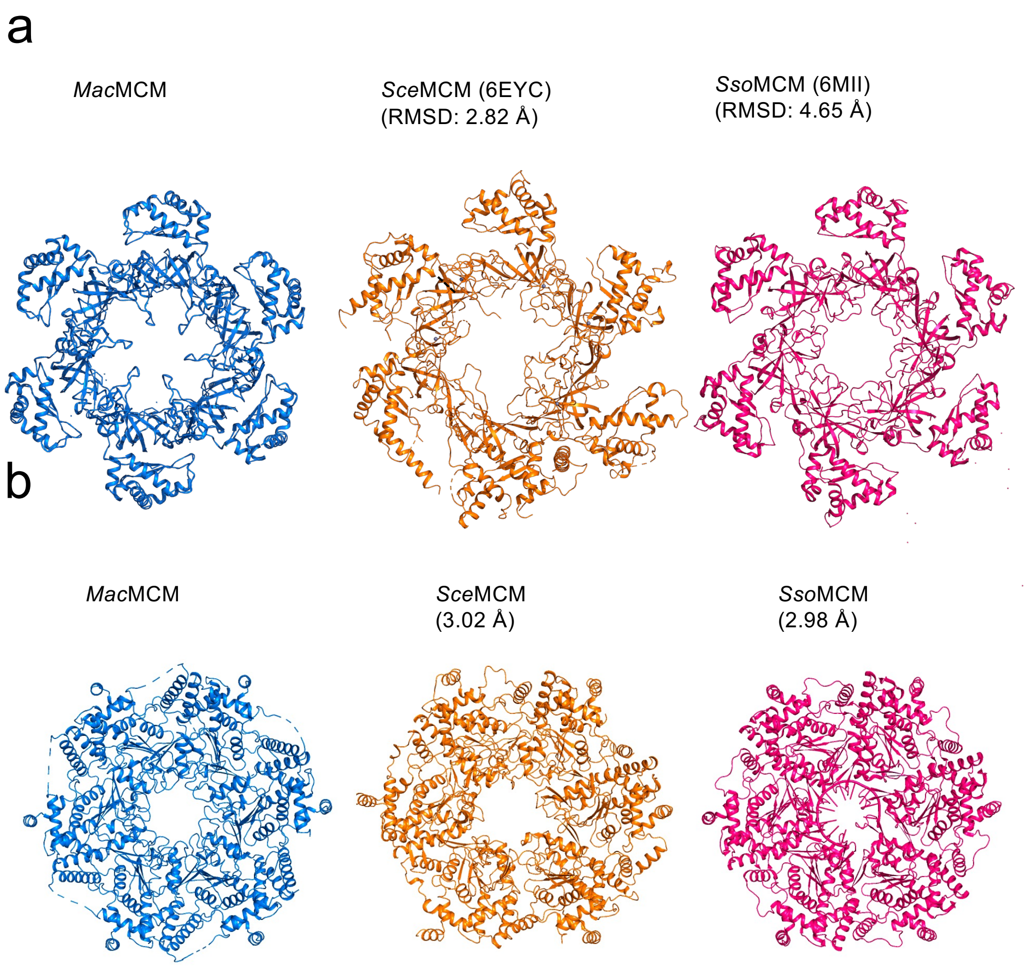

Supplementary Figure 14: Comparison of *Mac*MCM with other MCM hexamer structures. The structure of *Mac*MCM hexamer was compared against *Sce*MCM (PDB: 6EYC) and *Sso*MCM in PyMol. As the N- and C-terminal tiers are somewhat flexible in positioning, each MCM was split into its constituent N-terminal, **a** and C-terminal tiers **b,** and then structurally aligned to the *Mac*MCM structure. The all-atom RMSD for each tier is shown for each alignment.

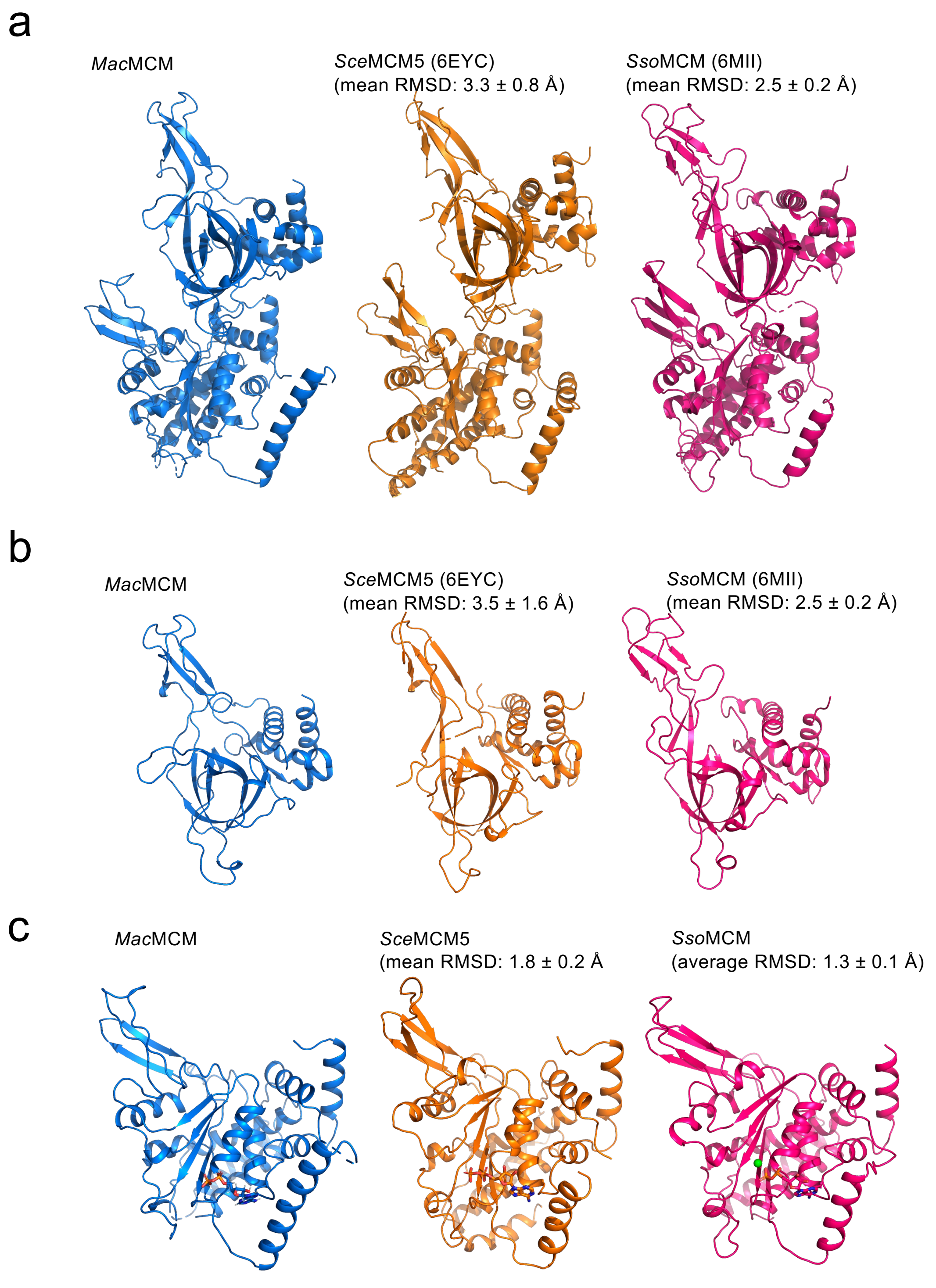

Supplementary Figure 15: Structural diversity of subunits and subdomains. The structure of each MCM (*Mac*MCM: PDB:8Q67, *Sce*MCM: PDB: 6EYC, *Sso*MCM: PDB: 6MII) was split into different groups that compared each: subunit, **a**, N-terminal domain, **b**, or C-terminal ,**c**,. Each structure in a group was then structurally aligned aligned to every other structure in the group to give the mean all-atom RMSD as shown, above an example for each alignment visualised in PyMol,

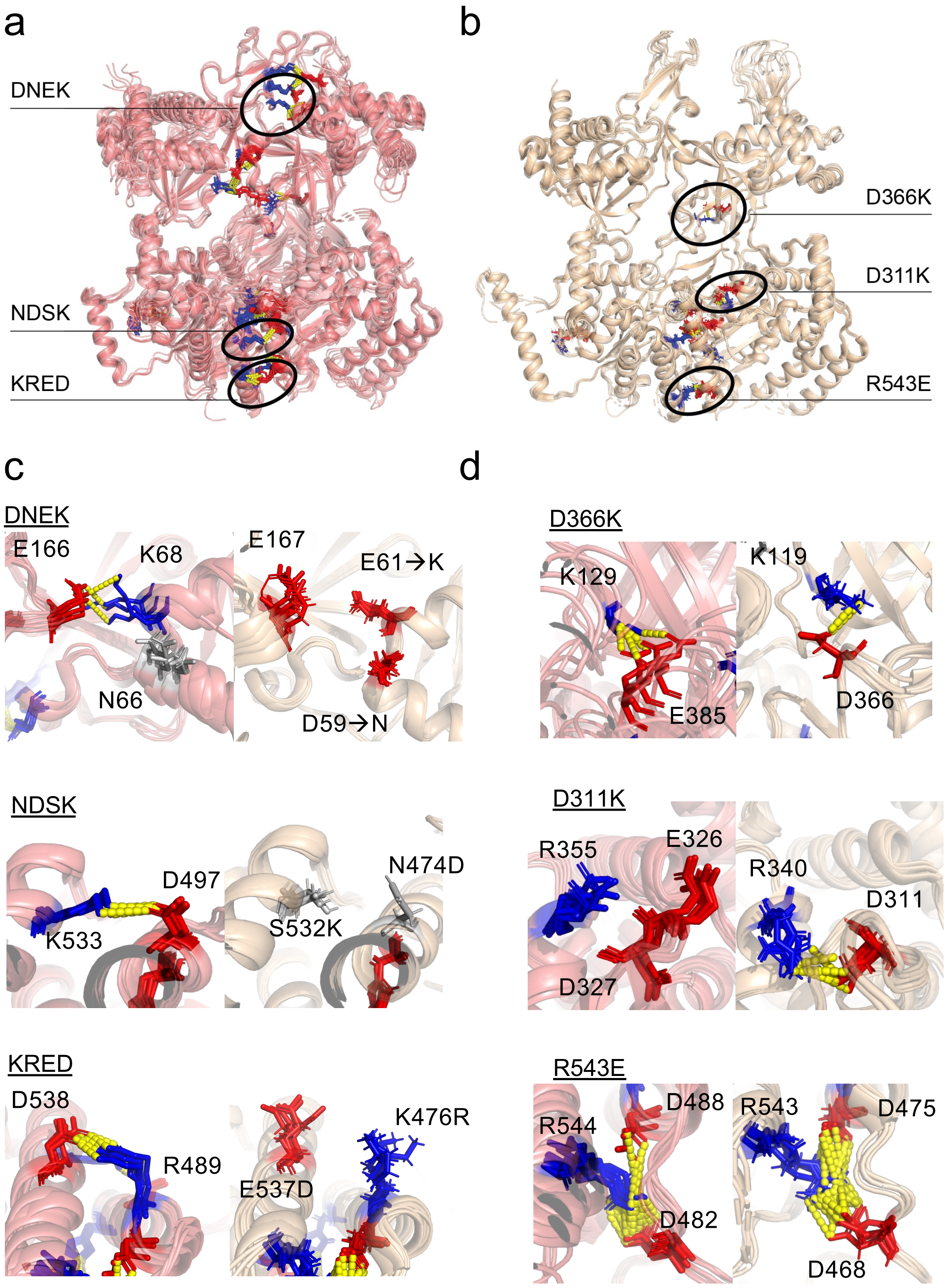

Supplementary Figure 16: Salt Bridge Mutant Design Rationale. a-b View of structurally aligned subunit pairs. Each protein is represented in the cartoon format. Ligands are represented in the stick format. Residues that putatively participate in a salt-bridge pair are shown in the stick format and coloured depending whether the amino acid sidechain is acidic (red) or basic (blue). Salt bridges that have been positively identified are shown with a dotted yellow line. **a** Sites which are re-engineered into *Mac*MCM (plus-SB) from *Sso*MCM are shown on the *Sso*MCM structure. **b** Salt-bridges that are removed (minus-SB) are shown on the *Mac*MCM structure. **c-d** Each site modified in *Mac*MCM (right panels) are shown in greater detail with respect to *Sso*MCM (left panels). **c** Structural alignment was used to identify potential modification sites in *Mac*MCM to match the salt-bridge interaction network of *Sso*MCM. **d** *Mac*MCM Salt bridges subjected to knock-out mutation are shown.

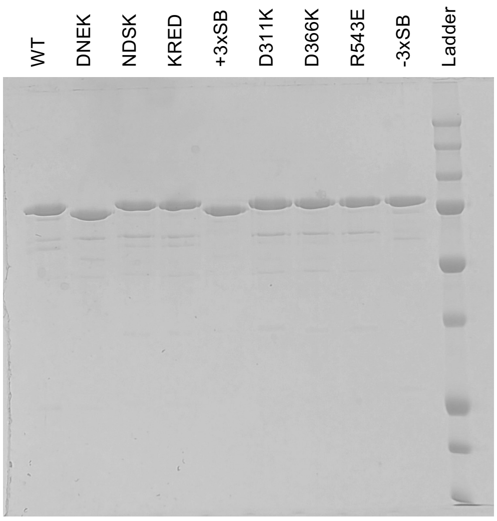

Supplementary Figure 17: Purification of salt bridge mutant MCM. Purity analysis of recombinant proteins used in this study. SDS-PAGE analysis of 1 µg each purified MCM.

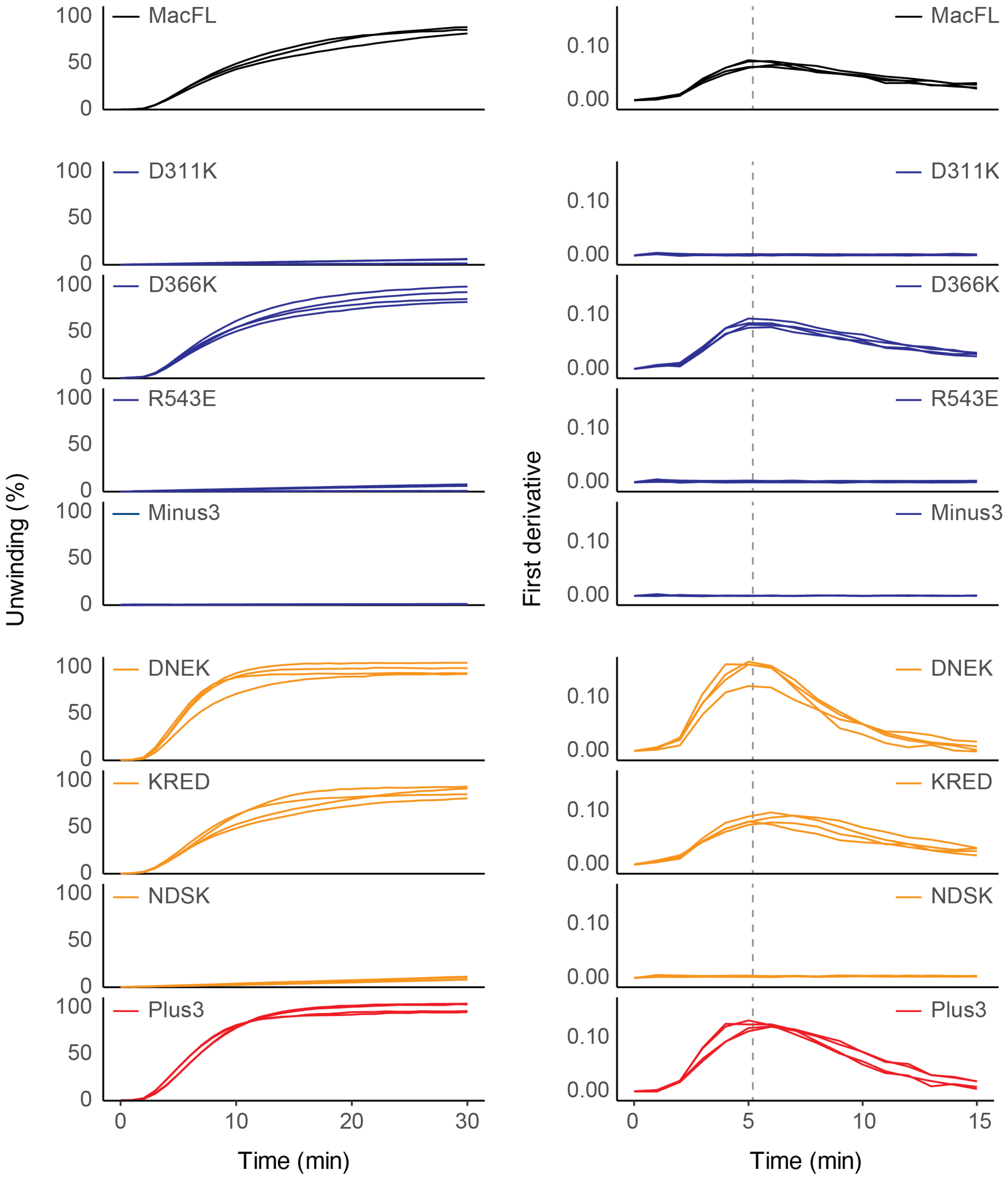

Supplementary Figure 18: DNA Unwinding Kinetics of Salt Bridge Mutants (Left) Real time DNA-unwinding curves for all purified *Mac*MCM and salt bridge variants at 25 ºC (N = 4); (Right) First derivative of unwinding curves fto show lag times

Supplementary references

1. Beeder, J., Nilsen, R. K., Rosnes, J. T., Torsvik, T. & Lien, T. Archaeoglobus fulgidus isolated from hot North Sea oil field waters. *Appl. Environ. Microbiol.* **60**, 1227–1231 (1994).

2. Sako, Y. *et al.* Aeropyrum pernix gen. nov., sp. nov., a novel aerobic hyperthermophilic archaeon growing at temperatures up to 100 degrees C. *Int. J. Syst. Bacteriol.* **46**, 1070–1077 (1996).

3. Robinson, J. L. *et al.* Growth kinetics of extremely halophilic archaea (family halobacteriaceae) as revealed by arrhenius plots. *J. Bacteriol.* **187**, 923–929 (2005).

4. Miller-Coleman, R. L. *et al.* Korarchaeota diversity, biogeography, and abundance in Yellowstone and Great Basin hot springs and ecological niche modeling based on machine learning. *PLoS One* **7**, e35964 (2012).

5. Golyshina, O. V. *et al.* The novel extremely acidophilic, cell-wall-deficient archaeon Cuniculiplasma divulgatum gen. nov., sp. nov. represents a new family, Cuniculiplasmataceae fam. nov., of the order Thermoplasmatales. *Int. J. Syst. Evol. Microbiol.* **66**, 332–340 (2016).

6. Jarvis, G. N. *et al.* Isolation and identification of ruminal methanogens from grazing cattle. *Curr. Microbiol.* **40**, 327–332 (2000).

7. Boone, D. R. *et al.* Isolation and Characterization of Methanohalophilus portucalensis sp. nov. and DNA Reassociation Study of the Genus Methanohalophilus. *Int. J. Syst. Bacteriol.* **43**, 430–437 (1993).

8. Kurr, M. *et al.* Methanopyrus kandleri, gen. and sp. nov. represents a novel group of hyperthermophilic methanogens, growing at 110°C. *Arch. Microbiol.* **156**, 239–247 (1991).

9. Liu, C. *et al.* Comparative proteomic analysis of Methanothermobacter thermautotrophicus reveals methane formation from H2 and CO2 under different temperature conditions. *Microbiologyopen* **8**, e00715 (2019).

10. Paper, W. *et al.* Ignicoccus hospitalis sp. nov., the host of "Nanoarchaeum equitans." *Int. J. Syst. Evol. Microbiol.* **57**, 803–808 (2007).

11. Qin, W. *et al.* Nitrosopumilus maritimus gen. nov., sp. nov., Nitrosopumilus cobalaminigenes sp. nov., Nitrosopumilus oxyclinae sp. nov., and Nitrosopumilus ureiphilus sp. nov., four marine ammonia-oxidizing archaea of the phylum Thaumarchaeota. *Int. J. Syst. Evol. Microbiol.* **67**, 5067–5079 (2017).

12. Weinberg, M. V., Schut, G. J., Brehm, S., Datta, S. & Adams, M. W. W. Cold shock of a hyperthermophilic archaeon: Pyrococcus furiosus exhibits multiple responses to a suboptimal growth temperature with a key role for membrane-bound glycoproteins. *J. Bacteriol.* **187**, 336–348 (2005).

13. Zaparty, M. *et al.* "Hot standards" for the thermoacidophilic archaeon Sulfolobus solfataricus. *Extremophiles* **14**, 119–142 (2010).

14. Mitchell, A. L. *et al.* InterPro in 2019: improving coverage, classification and access to protein sequence annotations. *Nucleic Acids Res.* **47**, D351–D360 (2018).

15. Sievers, F. *et al.* Fast, scalable generation of high-quality protein multiple sequence alignments using Clustal Omega. *Mol. Syst. Biol.* **7**, 539 (2011).

16. Meagher, M., Epling, L. B. & Enemark, E. J. DNA translocation mechanism of the MCM complex and implications for replication initiation. *Nat. Commun.* **10**, 3117 (2019).

17. Croll, T. I. ISOLDE: a physically realistic environment for model building into low-resolution electron-density maps. *Acta Crystallogr D Struct Biol* **74**, 519–530 (2018).
